# Existence and convergence of the length-preserving elastic flow of clamped curves

**DOI:** 10.1007/s00028-024-00988-1

**Published:** 2024-07-02

**Authors:** Fabian Rupp, Adrian Spener

**Affiliations:** 1 Faculty of Mathematics, University of Vienna, Oskar-Morgenstern-Platz 1, 1090 Vienna, Austria; 2 Institute of Applied Analysis, Ulm University, Helmholtzstraße 18, 89081 Ulm, Germany

**Keywords:** Nonlocal geometric evolution equation, Clamped boundary conditions, Elastic energy, Willmore functional, Łojasiewicz–Simon gradient inequality, Primary 53E40, Secondary 35K52, 35K55

## Abstract

We study the evolution of curves with fixed length and clamped boundary conditions moving by the negative $L^2$-gradient flow of the elastic energy. For any initial curve lying merely in the energy space we show existence and parabolic smoothing of the solution. Applying previous results on long-time existence and proving a constrained Łojasiewicz–Simon gradient inequality we furthermore show convergence to a critical point as time tends to infinity.

## Introduction and main results

For an immersed curve $f:I:=[0,1]\rightarrow {\mathbb {R}}^d$, $d\ge 2$, its *Euler–Bernoulli energy* or simply *elastic energy* is defined by

$$\begin{aligned} {\mathcal {E}}(f):=\frac{1}{2}\int \nolimits _{I} |\vec {\kappa }|^2\!\textrm{d}s. \end{aligned}$$

Here $\textrm{d}s:=\gamma \!\textrm{d}x$, where $\gamma :=|\partial _x f|$ denotes the *arc-length element*, and $\vec {\kappa }:=\partial _s^2 f$ is the *curvature vector field*, where $\partial _s :=\gamma ^{-1}\partial _x$ is the *arc-length derivative*.

In this article, we deform an initial curve $f_0$ in such a way that its elastic energy decreases as fast as possible, while keeping the *(total) length* ${\mathcal {L}}(f):=\int \nolimits _I\!\textrm{d}s$ fixed. This yields the geometric evolution equation

1.1 $$\begin{aligned} \partial _t^{\perp }f = - \nabla _s^2\vec {\kappa }-\frac{1}{2}|\vec {\kappa }|^2\vec {\kappa }+\lambda \vec {\kappa }. \end{aligned}$$

Here $\nabla _s$ denotes the connection on the normal bundle along *f*, i.e. $\nabla _s :=P^{\perp }\partial _s$, where $P^{\perp }X :=X^{\perp _f}:=X-\langle X,\partial _s f\rangle \partial _s f$ denotes the orthogonal projection along *f* of any vector field *X* along *f*. The *Lagrange multiplier* $\lambda$ depends on the solution *f* and is given by

1.2 $$\begin{aligned} \lambda (f) = \lambda (f)(t)= \frac{\int \nolimits _I \left\langle \nabla _s^2 \vec {\kappa }+ \frac{1}{2} |\vec {\kappa }| ^2 \vec {\kappa }, \vec {\kappa }\right\rangle \!\textrm{d}s}{\int \nolimits _I \vert \vec {\kappa }\vert ^{^2} \!\textrm{d}s}. \end{aligned}$$

Here $\langle \cdot , \cdot \rangle$ denotes the Euclidean inner product. Note that the evolution (1.1) is *geometric*, i.e. if a smooth *f* satisfies (1.1), then for any smooth family of reparametrizations $\Phi :[0,T)\times I\rightarrow I$ so does ${\hat{f}}(t,x) :=(f\circ \Phi )(t,x):=f(t, \Phi (t,x))$. In addition to the evolution (1.1), we prescribe *clamped boundary conditions*, fixing position and the unit tangent of the curve at the endpoints of *I*. For an immersed curve $f_0$ we hence study the following initial boundary value problem.

1.3 $$\begin{aligned} \left\{ \begin{array}{rll} \partial _tf &{}= - \nabla _s^2 \vec {\kappa }-\frac{1}{2} |\vec {\kappa }| ^2 \vec {\kappa }+ \lambda \vec {\kappa }+ \theta \partial _s f &{} \text { on } (0,T)\times {I} \\ f(0,x)&{}=f_0(x) &{} \text { for }x\in {I} \\ f(t,y)&{}=p_y &{} \text { for }0\le t<T, y\in \partial I \\ \partial _s f(t,y) &{}= \tau _y &{}\text { for } 0\le t < T, y\in \partial I,\\ \end{array} \right. \end{aligned}$$

where the unknown $\theta :[0,T)\times I\rightarrow {\mathbb {R}}$, $\theta = \langle \partial _t f, \partial _s f\rangle$ is the *tangential velocity*. By the integral representation of $\lambda$, (1.3) becomes a *nonlocal quasilinear system* which is also *degenerate* parabolic by its geometric nature. We assume that the boundary data $p_y\in {\mathbb {R}}^d, \tau _y\in {\mathbb {S}}^{d-1}\subset {\mathbb {R}}^d$ satisfy the *compatibility conditions*

1.4 $$\begin{aligned} f_0(y) = p_y \text { and } \partial _s f_0(y)=\tau _y \quad \text { for }y\in \partial I. \end{aligned}$$

Note that (1.3) is preserved under a smooth family of reparametrizations $\Phi$ which keeps the boundary $\partial I$ fixed, where the tangential velocity might change.

It is not difficult to see that $\lambda$ is chosen exactly in such a way that the length remains fixed during the flow, since along any sufficiently smooth solution of (1.3) we have

1.5 $$\begin{aligned} \frac{\!\textrm{d}}{\!\textrm{d}t} {\mathcal {L}}(f) = -\int \nolimits _I\langle \vec {\kappa }, \partial _t f\rangle \,\!\textrm{d}s = \int \nolimits _I \left\langle \nabla _s^2 \vec {\kappa }+ \frac{1}{2} |\vec {\kappa }| ^2 \vec {\kappa }, \vec {\kappa }\right\rangle \, \!\textrm{d}s - \lambda \int \nolimits _I |\vec {\kappa }|^2\,\!\textrm{d}s = 0, \end{aligned}$$

whereas the energy indeed decreases since by (1.5)

1.6 $$\begin{aligned} \frac{\!\textrm{d}}{\!\textrm{d}t} {\mathcal {E}}(f)&= \int \nolimits _I\langle \nabla {\mathcal {E}}(f), \partial _t f \rangle \,\!\textrm{d}s = \int \nolimits _I\langle \nabla {\mathcal {E}}(f) - \lambda \vec {\kappa }, \partial _t^{\perp } f \rangle \,\!\textrm{d}s = -\int \nolimits _I |\partial _t^{\perp } f|^2\,\!\textrm{d}s, \end{aligned}$$

using that the $L^2(\!\textrm{d}s_f)$-gradient of ${\mathcal {E}}$ is given by $\nabla {\mathcal {E}}(f) = \nabla _s^2 \vec {\kappa }+ \frac{1}{2} |\vec {\kappa }| ^2 \vec {\kappa }$. In the above calculations, we also used the fact that all boundary terms vanish due to the boundary conditions. In order for $\lambda$ to be well-defined, we need to ensure that $f(t):=f(t, \cdot )$ is not a piece of a straight line. This can be guaranteed with no restrictions on $\tau _0, \tau _1$ by requiring

1.7 $$\begin{aligned} |p_0-p_1| < \ell :={\mathcal {L}}(f_0), \end{aligned}$$

so ${\mathcal {E}}(f_0)>0$, see Sect. 2.1 for a more detailed analysis of $\lambda$.

In [10], long-time existence for smooth solutions of (1.3) with tangential velocity $\theta \equiv 0$ under assumption (1.7) was shown with the help of interpolation inequalities. For the short-time existence the authors of [10] refer to the beginning of Section 3 in [17], where the short-time existence in the setting of Hölder spaces is only sketched for the case of closed curves. Moreover, the uniform bounds in [10, Theorem 1.1] imply subconvergence after reparametrization as $t\rightarrow \infty$. However, different sequences could still have different limits.

The contribution of this paper is twofold: First, we give a rigorous and fairly concise proof of short-time existence and parabolic smoothing for the elastic flow (1.3). Compared to the previous classical existence results for elastic flows, where the initial datum is assumed to be smooth [10, 17, 42] or at least with Hölder continuous second derivative [50], one major improvement is that we allow for rough initial values, lying merely in the natural *energy space*, see Remark 2.11 for a detailed discussion. In contrast to existence theorems relying on the minimizing movement scheme (cf. [5, 6, 33, 39, 41]), our methods rely on *maximal regularity*, yielding here smooth solutions, cf. Theorem 1.1 below, while still allowing for rough initial data. The price for this substantial improvement is that the necessary contraction estimates become quite technical and rely delicately on the precise structure of (1.3). This is the first existence result for an elastic flow with general initial data of such weak regularity.

### Theorem 1.1

Let $f_0\in W^{2,2}(I;{\mathbb {R}}^d)$ be immersed, let ${p_0, p_1\in {\mathbb {R}}^d}$ and $\tau _0, \tau _1 \in {\mathbb {S}}^{d-1}$ satisfy (1.4) and (1.7). Then, there exist $T\!>\!0$ and a solution ${f\!\in \! W^{1,2}(0,T;L^2}{(I,{\mathbb {R}}^d))}{\cap L^2(0,T;W^{4,2}(I;{\mathbb {R}}^d))}$ of (1.3).

Moreover, we show that under the assumptions (1.4) and (1.7), the solution in Theorem 1.1 instantaneously becomes smooth, both in space and time, cf. Theorem 3.1.

Secondly we prove and apply a *constrained Łojasiewicz–Simon gradient inequality* (cf. [45]) to deduce convergence of the flow, where a new estimate (see Lemma 4.10) substantially simplifies the argument for the convergence result compared to previous works, cf. [8, 13].

### Theorem 1.2

Let $f_0\in W^{2,2}(I;{\mathbb {R}}^d)$ be an immersed curve and suppose ${p_0, p_1\in {\mathbb {R}}^d}$ and $\tau _0, \tau _1 \in {\mathbb {S}}^{d-1}$ satisfy (1.4) and (1.7). Then, there exists a smooth family of curves $f:(0,\infty )\times I\rightarrow {\mathbb {R}}^d$ solving (1.3), such that

(i) $$f(t)\rightarrow f_0$$ in $$W^{2,2}(I;{\mathbb {R}}^d)$$ as $$t\rightarrow 0$$;
(ii) $f(t)\rightarrow f_{\infty }$ smoothly after reparametrization as $t\rightarrow \infty$, where $f_\infty$ is a constrained clamped elastica, i.e. a solution of
  1.8 $$\begin{aligned} \left\{ \begin{array}{rll} - \nabla _s^2 \vec {\kappa }-\frac{1}{2} |\vec {\kappa }| ^2 \vec {\kappa }+ \lambda \vec {\kappa }&{}= 0&{} \text { on } I\\ f(y)&{}=p_y &{}\text { for }y\in \partial I \\ \partial _s f(y) &{}= \tau _y &{}\text { for } y\in \partial I \end{array} \right. \end{aligned}$$
  for some $$\lambda \in {\mathbb {R}}$$.

Together with the previously mentioned work [10] this paper completes the study of the existence and convergence of the elastic flow of clamped curves with fixed length. Unfortunately, due to the low regularity of the initial curves considered here, we are not able to show uniqueness for the solution of the geometric evolution equation (1.3).

In the smooth category, one can show uniqueness “up to reparametrization” by a PDE argument similar to [22]. However, due to our low regularity we were not able to prove sufficient contraction estimates. The reason for that is the rigid characterization of Lipschitz properties of Nemytskii operators, see for instance [4, Theorem 3.10, Theorem 7.9].

The elastic energy of curves has already been studied by Bernoulli. The analysis of the elastic flow, i.e. the one-dimensional analogue of the Willmore flow, started with [42] and [17]. The boundary value problem for the elastic flow was considered in [25] for clamped curves and in [9] for natural second-order boundary conditions, see also [26, 27, 52] for related second-order evolutions. For further related literature on elastic flows, we refer to [5, 6, 35, 39, 41, 50]. Recent research has also studied the geometric evolution of networks and previously achieved results were applied to the elastic flow of networks, see e.g. [11, 15, 20, 21, 37]. Moreover, the elastic flow with different ambient geometries has been considered in [14, 34, 43], especially, the case of hyperbolic space [34] is of interest, cf. [12, 24]. Additionally, we mention the elastic flow of closed curves under a length and area constraint [38].

The Łojasiewicz–Simon gradient inequality is a remarkable result on *(real) analytic* functions which was first proven in ${\mathbb {R}}^d$ [28] and later generalized to infinite dimensions [49], see also [7]. Nowadays, it is the fundamental tool for investigating the asymptotic properties of gradient flows with analytic energies, which has been used for many geometric evolution equations, see for instance [8, 13, 19, 30, 31, 40, 47, 48] and also [36] for a different approach. The fixed-length constraint in (1.3) and (1.5) obstructs the use of [7] to deduce the gradient inequality, which is why we apply a recent extension to constrained energies [45]. We emphasize that this article is the first application of the constrained Łojasiewicz–Simon gradient inequality for a constrained gradient flow.

This article is structured as follows. In Sect. 2, we pick a specific tangential velocity such that (1.3) becomes a parabolic system, which we reduce to a fixed point equation. The existence of a fixed point is then established on a small time interval, using the concept of maximal $L^p$-regularity together with appropriate contraction estimates. Section 3 is devoted to show instantaneous smoothing of our solution, both in space and in time. After that, we prove long-time existence and a refined Łojasiewicz–Simon gradient inequality to finally prove Theorem 1.2 in Sect. 4. For the sake of readability, some details on the contraction estimates and the parabolic smoothing have been moved to the appendix or can be found in the first author’s dissertation [46, Chapter 3].

## Short-time existence

The goal of this section is to prove Theorem 1.1. As in [20], we prescribe an explicit tangential motion to transform (1.3) into a quasilinear parabolic system. We then perform a linearization and use the theory of maximal $L^p$-regularity and suitable contraction estimates to prove Theorem 1.1 using a fixed point argument. We consider an initial datum merely lying in $W^{2,2}_{Imm}(I;{\mathbb {R}}^d)$, the space of $W^{2,2}$-immersions. This is a natural space for the elastic energy, since it is the roughest Sobolev space where ${\mathcal {E}}$ remains finite.

### On the Lagrange multiplier

To ensure that the Lagrange multiplier is well-defined, one needs to prevent the denominator from vanishing. Write $\lambda (f) =:\frac{N(f)}{2{\mathcal {E}}(f)}$, where *N*(*f*) denotes the numerator in (1.2) and observe that for a solution of (1.3) we have

$$\begin{aligned} |f(t,0)-f(t,1)| = |p_0-p_1| <\ell ={\mathcal {L}}(f(t)) \quad \text { for all }t\in [0,T), \end{aligned}$$

using the boundary conditions, (1.7) and (1.5). In particular, *f*(*t*) cannot be part of a straight line, so ${\mathcal {E}}(f(t))>0$ for all $t\in [0,T)$. Moreover, we observe that after integration by parts we have

2.1 $$\begin{aligned} N(f) =\int \nolimits _I\langle \nabla {\mathcal {E}}(f), \vec {\kappa }\rangle \,\!\textrm{d}s = \langle \nabla _s \vec {\kappa }, \vec {\kappa }\rangle |_{\partial I} - \int \nolimits _I |\nabla _s \vec {\kappa }|^2 \,\!\textrm{d}s + \frac{1}{2} \int \nolimits _I |\vec {\kappa }|^4 \,\!\textrm{d}s. \end{aligned}$$

Note that in (2.1), no derivatives of second order of the curvature appear, which means that the Lagrange multiplier is formally of lower order compared to $\nabla {\mathcal {E}}(f)$. This is extremely useful later on, since we can rely on the well-studied property of maximal $L^p$-regularity for a local operator in the linearization and treat the Lagrange multiplier as a nonlinearity in the fixed point argument.

### From the geometric problem to a quasilinear PDE

As a next step, we explicitly compute the right-hand side of (1.1). By Proposition A.1

$$\begin{aligned} \nabla {\mathcal {E}}(f)&= \nabla _s^2 \vec {\kappa }+\frac{1}{2} |\vec {\kappa }| ^2 \vec {\kappa }={\mathcal {A}}(f)^{\perp }, \end{aligned}$$

where

2.2 $$\begin{aligned} {\mathcal {A}}(f)&:=\frac{\partial _x^{4}f}{\gamma ^{4}} -6 \frac{\langle \partial _{x}^{2} f, \partial _x f\rangle }{\gamma ^{6}}\partial _x^{3}f - 4 \frac{\langle \partial _x^{3}f, \partial _x f\rangle }{\gamma ^{6}}\partial _x^{2}f - \frac{5}{2} \frac{|\partial _x^{2} f|^{2}}{\gamma ^{6}} \partial _x^{2} f \nonumber \\&\quad + \frac{35}{2} \frac{\langle \partial _x^{2}f, \partial _x f\rangle ^{2}}{\gamma ^{8}} \partial _x^{2}f\nonumber \\&=:\frac{\partial _x^{4}f}{\gamma ^{4}} +{\tilde{F}}(\gamma ^{-1}, \partial _x f, \partial _x^2 f, \partial _x^3 f). \end{aligned}$$

In order to solve (1.3), we study the following evolution problem, prescribing an explicit tangential motion $\theta =\mu$ to make the problem parabolic. We want to find a family of immersions $f:[0,T)\times I\rightarrow {\mathbb {R}}^d$ satisfying

2.3 $$\begin{aligned} \left\{ \begin{array}{rll} \partial _t f &{}= - \nabla _s^2 \vec {\kappa }-\frac{1}{2} |\vec {\kappa }| ^2 \vec {\kappa }+ \mu \partial _s f + \lambda \vec {\kappa }&{} \text { on } (0,T)\times {I} \\ f(0,x)&{}=f_0(x) &{} \text { for }x\in {I} \\ f(t,y)&{}=p_y &{} \text { for }0\le t<T,y\in \partial I \\ \partial _{x} f(t,y)&{}= \tau _y{\gamma _0(y)} &{} \text { for }0\le t < T,y\in \partial I.\\ \end{array}\right. \end{aligned}$$

with $\lambda$ as in (1.2) and $\mu =\mu (f):[0,T)\times I\rightarrow {\mathbb {R}}$ given by $\mu :=- \langle {\mathcal {A}}(f), \partial _s f\rangle$. Note that the first-order boundary conditions are a linear version of the general boundary conditions in (1.3) and thus easier to handle. The system (2.3) is often referred to as the *analytic problem*.

For $1<p<\infty$ and $T>0$, we consider the space of solutions

$$\begin{aligned} {\mathbb {X}}_{T,p} :=W^{1,p}\left( 0,T; L^p(I;{\mathbb {R}}^d)\right) \cap L^p\left( 0,T; W^{4,p}(I;{\mathbb {R}}^d)\right) \end{aligned}$$

and the space of data

$$\begin{aligned} {\mathbb {Y}}_{T,p}^1 :=L^p\left( 0,T;L^p(I;{\mathbb {R}}^d)\right) . \end{aligned}$$

The space of initial data is given by the *Besov space*

$$\begin{aligned} {\mathbb {Y}}_p^{2}:=\{ f(0) \mid f\in {\mathbb {X}}_{T,p}\} = B^{4(1-\frac{1}{p})}_{p,p}(I;{\mathbb {R}}^d), \end{aligned}$$

see for instance [16, Section 2]. We also consider the solution space with vanishing trace at time $t=0$ given by

$$\begin{aligned} {}_{0}{{\mathbb {X}}}_{T,p} :=\{ f\in {\mathbb {X}}_{T,p}\mid f(0)=0\}. \end{aligned}$$

For convenience, we also set ${\mathbb {Y}}_{T,p} :={\mathbb {Y}}_{T,p}^1 \times {\mathbb {Y}}_p^2$.

### Linearization of the analytic problem

If we linearize (2.3) for $\lambda \equiv 0$, we obtain a linear parabolic system. This system is a local PDE which we can apply maximal regularity theory to. First, assuming $\lambda \equiv 0$ and using (2.2), the evolution in (2.3) has the form

$$\begin{aligned} \partial _t f = -{\mathcal {A}}(f) =:-\frac{\partial _x^{4}f}{\gamma ^{4}} - {\tilde{F}}(\gamma ^{-1},\partial _xf, \partial _{x}^{2}f, \partial _x^{3}f) \end{aligned}$$

with ${\mathcal {A}}$ as in (2.2). If we freeze coefficients for the highest order term at the initial datum $f_0$ we get

2.4 $$\begin{aligned} \partial _t f + \frac{\partial _x^{4}f}{{\gamma _0}^{4}}&= \left( \frac{1}{\gamma _0^{4}}-\frac{1}{\gamma ^{4}}\right) \partial _x^{4} f- {\tilde{F}}(\gamma ^{-1},\partial _xf, \partial _{x}^{2}f, \partial _x^{3}f)\nonumber \\&=:F(\gamma ^{-1},\partial _x f, \partial _x^{2}f, \partial _x^{3}f, \partial _x^{4}f), \end{aligned}$$

where $\gamma _0 :=\gamma (0, \cdot ) = |\partial _x f_0|$ and ${\tilde{F}}$ is as in (2.2). The linearized system we associate to (2.3) with $\lambda \equiv 0$ is

2.5 $$\begin{aligned} \left\{ \begin{array}{rll} \partial _t f + \frac{1}{\gamma _0^{4}}\partial _x^{4}f &{}= F&{} \text { on } (0,T)\times {I} \\ f(0,x)&{}=f_0(x) &{} \text { for }x\in {I} \\ f(t,y)&{}=p_y &{} \text { for }0\le t<T,y\in \partial I \\ {\partial _x f(t,y)}&{}= \tau _y{\gamma _0(y)} &{} \text { for }0\le t < T,y\in \partial I.\\ \end{array}\right. \end{aligned}$$

We can now apply the general $L^p$-theory for parabolic systems to obtain the following classical maximal regularity result, whose proof can be found in [46, Chapter 3, Section 2.3]. For the definition of the spaces for the boundary data ${\mathcal {D}}^{i}_{T,p}$ with $i=0,1,$ see (B.2).

#### Theorem 2.1

Let $p\in (\frac{5}{3},\infty )$, $0<T\le T_0$. Suppose $a\in {\mathcal {C}}([0,T_0]\times I;{\mathbb {R}})$ such that $a(t,x)\ge \alpha$ for some $\alpha >0$ and all $t\in [0,T_0], x\in I$. Let $(\psi ,f_0) \in {\mathbb {Y}}_{T,p}$, $b^0 \in {\mathcal {D}}^0_{T,p}$ and $b^1\in {\mathcal {D}}^1_{T,p}$ such that the following compatibility conditions are satisfied:

2.6 $$\begin{aligned} b^0(0,y)&= f_0(y)&\text {for }y\in \partial I,\nonumber \\ b^1(0,y)&= \partial _x f_0(y)&\text {for }y\in \partial I. \end{aligned}$$

Then, there exists a unique $f\in {\mathbb {X}}_{T,p}$ such that

2.7 $$\begin{aligned} \left\{ \begin{array}{rll} \partial _t f + a\partial _x^{4}f &{}= \psi &{} \text { on } (0,T)\times {I} \\ f(0,x)&{}=f_0(x) &{} \text { for }x\in {I} \\ f(t,y)&{}=b^0(t,y) &{} \text { for }0\le t<T,y\in \partial I \\ \partial _x f(t,y)&{}= b^1(t,y) &{} \text { for }0\le t < T,y\in \partial I,\\ \end{array}\right. \end{aligned}$$

and there exists $C=C(p, T, a)>0$ such that

2.8 $$\begin{aligned} \Vert f \Vert _{{\mathbb {X}}_{T,p}} \le C\left( \Vert \psi \Vert _{{\mathbb {Y}}_{T,p}^1} + \Vert f_0 \Vert _{{\mathbb {Y}}_{p}^2} + \Vert b^0 \Vert _{{\mathcal {D}}^0_{T,p}} + \Vert b^1 \Vert _{{\mathcal {D}}^1_{T,p}}\right) . \end{aligned}$$

Moreover, if $b^0=0$ and $b^1=0$, then we may choose $C=C(p,T_0,a)$ independent of $T\le T_0$.

Now, we want to solve (2.3) for initial data $f_0\in W^{2,2}(I;{\mathbb {R}}^d)$ using a fixed point argument. Note that $B^{2}_{2,2}(I;{\mathbb {R}}^d)=W^{2,2}(I;{\mathbb {R}}^d)$ by (B.1), so $p=2$ is a fine setup to deal with the desired initial data, see Remark 2.11 for a more detailed discussion. We observe that the linearized system (2.5) can be viewed as a special case of Theorem 2.1 with $a= \frac{1}{\gamma _0^4}$, $b^0 = (p_0, p_1)$, $b^1 = (\tau _0, \tau _1)$ and $\psi =F$.

Throughout the rest of this section, we exclusively work with $p= 2$. To simplify notation the spaces ${\mathbb {X}}_{T}, {\mathbb {Y}}_T, {\mathcal {D}}^0, {\mathcal {D}}^1$ denote the respective spaces with $p=2$.

### Contraction estimates

The key ingredient in the proof of the short-time existence is a contraction estimate for the nonlinearity in (2.3). We fix an initial datum $f_0\in W^{2,2}_{Imm}(I;{\mathbb {R}}^d)$ and boundary conditions $p_0, p_1\in {\mathbb {R}}^d$ and $\tau _0, \tau _1\in {\mathbb {S}}^{d-1}$ satisfying (1.4) and (1.7). For a *reference flow* ${\bar{f}}\in {\mathbb {X}}_{T=1}$ with ${\bar{f}}(0)=f_0$, and some *M* and $T\in (0,1]$ we define

2.9 $$\begin{aligned} {{\bar{B}}_{T,M}:=\left\{ f \in {\mathbb {X}}_T \mid f(0)=f_0 \text { and } \Vert f-{\bar{f}} \Vert _{{\mathbb {X}}_T}\le M\right\} .} \end{aligned}$$

We denote by *T* the *existence time* and by *M* the *contraction radius.* Since we take $T,M>0$ small later on, it is no restriction to only consider $T,M\le 1$. Later, we choose a specific reference flow ${\bar{f}}$, see Definition 2.5.

First, the following lemma yields uniform bounds from below on the arc-length element and the elastic energy for small times, ensuring that the system (2.3) does not immediately become singular. A detailed proof can be found in [46, Chapter 3, Section 2.4.1].

#### Lemma 2.2

For $T=T({\bar{f}})\in (0,1]$ small enough and $M\in (0, 1]$, any $f\in {{\bar{B}}_{T,M}}$ satisfies $\gamma (t,x)\ge \inf _{I}\frac{\gamma _0}{2}$ for all $(t,x)\in [0,T)\times I$. In particular, all curves $f(t,\cdot )$ are immersed.

#### Lemma 2.3

For $T=T({\bar{f}})\in (0,1]$ small enough and $M\in (0,1]$, any $f\in {{\bar{B}}_{T,M}}$ satisfies ${\mathcal {E}}(f(t)) \ge \frac{{\mathcal {E}}(f_0)}{3}>0$ (cf. (1.7)) for all $t\in [0,T)$,.

We now state the crucial contraction property of the nonlinearities. Since the space of initial data is the energy space, cf. Remark 2.11, the necessary estimates are quite involved and rely on the special structure of (1.1). For the sake of readability, some of the details and the proof of the following lemma are moved to Appendix C.

#### Lemma 2.4

Let $q\in (0,1)$. Then the following maps

$$\begin{aligned} \begin{aligned} {\mathcal {F}}:{{\bar{B}}_{T,M}} \rightarrow {\mathbb {Y}}_T^1,\quad {\mathcal {F}}(f)&:=F(\gamma ^{-1}, \partial _x f, \partial _x^2 f, \partial _x^3 f, \partial _x^4f) \\ \Lambda :{{\bar{B}}_{T,M}} \rightarrow {\mathbb {Y}}_T^1,\quad \Lambda (f)&:=\lambda (f)\vec {\kappa }_f\\ {\mathcal {N}}:{{\bar{B}}_{T,M}} \rightarrow {\mathbb {Y}}_T^1, \quad {\mathcal {N}}(f)&:={\mathcal {F}}(f)+\Lambda (f), \end{aligned} \end{aligned}$$

are well-defined *q*-contractions (i.e. Lipschitz continuous with Lipschitz constant *q*) for $T=T(q,{\bar{f}})$, $M=M(q, {\bar{f}})\in (0,1]$ small enough, with *F* as in (2.4) and $\lambda$ as in (1.2).

### The fixed point argument

We now reduce the analytic problem (2.3) to a fixed point equation and show local existence and uniqueness via the contraction principle. To that end, we first choose a specific reference solution ${\bar{f}}$ in (2.9) on the time interval $[0,1]\supset [0,T]$ for $0<T\le 1$.

#### Definition 2.5

We define the *reference solution* ${\bar{f}}$ to be the unique solution of the following initial boundary value problem.

$$\begin{aligned} \left\{ \begin{array}{rll} \partial _t {\bar{f}} + \frac{\partial _x^{4}{\bar{f}}}{\gamma _0^4} &{}= 0 &{} \text { on } {[0,1)}\times {I} \\ {\bar{f}}(0,x)&{}=f_0(x) &{} \text { for }x\in {I} \\ {\bar{f}}(t,y)&{}=p_y &{} \text { for }0\le t<{1}, y\in \partial I \\ \partial _x {\bar{f}}(t,y)&{}= \tau _y {\gamma _0(y)} &{}\text { for } 0\le t < {1},y\in \partial I.\\ \end{array}\right. \end{aligned}$$

Existence and uniqueness in the class

$$\begin{aligned} W^{1,2}\left( 0,{1}; L^2(I;{\mathbb {R}}^d)\right) \cap L^2\left( 0,{1}; W^{4,2}(I;{\mathbb {R}}^d)\right) \end{aligned}$$

follows from Theorem 2.1. Note that the restriction of the solution to any time interval [0, *T*] is the unique solution in the class ${\mathbb {X}}_{T}$ for all $0<T\le {1}$.

Fix $q\in (0,1)$ and take ${T}={T}(q,{\bar{f}})\in (0,1],M=M(q,{\bar{f}})\in (0,1]$ small enough such that Lemmas 2.2 to 2.4 hold. Let $f\in {{\bar{B}}_{T,M}}$. Then, we have ${\mathcal {N}}(f) \in {\mathbb {Y}}_{{T}}^{1}$, cf. Lemma 2.4. For $\psi :={\mathcal {N}}(f), b^0 :=(p_0,p_1)$, $b^1 :=(\tau _0, \tau _1)$, $a:=\gamma _0^{-4}\in {\mathcal {C}}([0,{1}]\times I)$ the compatibility conditions (2.6) are satisfied, since by (1.4) we have

$$\begin{aligned} b^0(0,y)&= f_0(y)&\text {for }y\in \partial I, \\ b^1(0,y)&= \tau _y {\gamma _0(y)} = \partial _x f_0(y)&\text {for }y\in \partial I. \end{aligned}$$

Hence, by Theorem 2.1, there exists a unique solution $g\in {\mathbb {X}}_T$ of the linear initial boundary value problem

2.10 $$\begin{aligned} \left\{ \begin{array}{rll} \partial _t g + \frac{\partial _x^{4}g}{\gamma _0^4} &{}= {\mathcal {N}}(f) &{} \text { on } (0,T)\times {I} \\ g(0,x)&{}=f_0(x) &{} \text { for }x\in {I} \\ g(t,y)&{}=p_y &{} \text { for } 0\le t<T,y\in \partial I \\ \partial _x g(t,y) &{}= \tau _y {\gamma _0(y)} &{} \text { for }0\le t < T,y\in \partial I.\\ \end{array}\right. \end{aligned}$$

#### Definition 2.6

We define the map $\Phi :{{\bar{B}}_{T,M}} \rightarrow {\mathbb {X}}_T, \Phi (f):=g$, where $g\in {\mathbb {X}}_T$ is the unique solution to (2.10).

#### Remark 2.7

Finding a solution of (2.3) in the ball ${{\bar{B}}_{T,M}} \subset {\mathbb {X}}_T$ is equivalent to finding a fixed point of the map $\Phi$ in Definition 2.6.

We now show that $\Phi$ is a contraction on ${{\bar{B}}_{T,M}}$ for $T,M>0$ small enough.

#### Proposition 2.8

Let $q\in (0,1)$. Then there exist $M=M(q,{\bar{f}})\in (0,1]$, $T=T(q,M,{\bar{f}})\in (0,1]$ such that $\Phi :{{\bar{B}}_{T,M}} \rightarrow {{\bar{B}}_{T,M}}$ is well-defined and a *q*-contraction, i.e.

2.11 $$\begin{aligned} \Vert \Phi (f)-\Phi ({\tilde{f}}) \Vert _{{\mathbb {X}}_T}\le q \Vert f-{\tilde{f}} \Vert _{{\mathbb {X}}_T} \end{aligned}$$

for all $$f,{\tilde{f}} \in {{\bar{B}}_{T,M}}$$.

#### Proof

The contraction property: Let $q\in (0,1)$ and $f, {\tilde{f}}\in {{\bar{B}}_{T,M}}$ and let $g=\Phi (f)$, ${\tilde{g}}=\Phi ({\tilde{f}})$. We observe that $g-{\tilde{g}}$ vanishes at $t=0$ and at the boundary $\partial I$ up to first order. Hence, by Definition 2.6 and (2.8), for some $C=C(f_0)=C({\bar{f}})>0$, independent of $T\in (0,1]$, we have

2.12 $$\begin{aligned} \Vert g-{\tilde{g}} \Vert _{{\mathbb {X}}_T}&\le C \Vert {\mathcal {N}}(f)-{\mathcal {N}}({\tilde{f}}) \Vert _{L^2(0,T;L^{2})}. \end{aligned}$$

Taking $T=T(q,{\bar{f}}),M=M(q,{\bar{f}})\in (0,1]$ small enough so that Lemma 2.4 can be applied with *q* replaced by *q*/(2*C*), we have

2.13 $$\begin{aligned} \Vert {\mathcal {N}}(f)-{\mathcal {N}}({\tilde{f}}) \Vert _{L^2(0,T;L^2)}\le \frac{q}{2C} \Vert f- {\tilde{f}} \Vert _{{\mathbb {X}}_T}. \end{aligned}$$

Equations (2.12) and (2.13) imply (2.11).

Well-definedness: Let $f\in {{\bar{B}}_{T,M}, g=\Phi (f)}$. Again by (2.8) we find

2.14 $$\begin{aligned} \Vert g-{\bar{f}} \Vert _{{\mathbb {X}}_T}&\le C \Vert {\mathcal {N}}(f)-0 \Vert _{L^2(0,T;L^{2})}\nonumber \\&\le C \left( \Vert {\mathcal {N}}(f)-{\mathcal {N}}({\bar{f}}) \Vert _{L^2(0,T;L^2)} + \Vert {\mathcal {N}}({\bar{f}}) \Vert _{L^2(0,T;L^{2})} \right) \nonumber \\&\le \frac{q}{2}\Vert f-{\bar{f}} \Vert _{{\mathbb {X}}_T} + C \Vert {\mathcal {N}}({\bar{f}}) \Vert _{L^2(0,T;L^{2})}\nonumber \\&\le \frac{q}{2}M + C\Vert {\mathcal {N}}({\bar{f}}) \Vert _{L^2(0,T;L^{2})}, \end{aligned}$$

where we applied (2.13) with ${\tilde{f}}={\bar{f}}$ in the third step. Now, by dominated convergence we have $\Vert {\mathcal {N}}({\bar{f}}) \Vert _{L^2(0,T;L^2)} \le \frac{M}{2C}$ reducing $T=T(q,M, {\bar{f}})\in (0,1]$ if necessary. Then, from (2.14) we conclude $\Vert \Phi (f)-{\bar{f}} \Vert _{{\mathbb {X}}_T}\le M$. $\square$

#### Theorem 2.9

Let $f_0\in W^{2,2}_{Imm}(I;{\mathbb {R}}^{d})$, $p_0, p_1\in {\mathbb {R}}^d$, $\tau _0, \tau _1\in {\mathbb {S}}^{d-1}$ satisfying (1.4) and (1.7). Then there exist $M>0$ and $T>0$ such that the system (2.3) has a unique solution $f\in {{\bar{B}}_{T,M}}\subset W^{1,2}\left( 0,T; L^2(I;{\mathbb {R}}^d)\right) \cap L^2\left( 0,T; W^{4,2}(I;{\mathbb {R}}^d)\right)$.

#### Proof

For $T,M>0$ as in Proposition 2.8 with $q=\frac{1}{2}$, the map $\Phi :{{\bar{B}}_{T,M}}\rightarrow {{\bar{B}}_{T,M}}$ is a contraction in the complete metric space ${{\bar{B}}_{T,M}}$ and hence has a unique fixed point $f\in {{\bar{B}}_{T,M}}$ by the contraction principle. Since any fixed point of $\Phi$ is a solution of (2.3) in ${{\bar{B}}_{T,M}}$ and vice versa, the claim follows. $\square$

#### Remark 2.10

By the construction of our solution and Lemma 2.2 and Lemma 2.3 the arc-length element $|\partial _x f|$ and the elastic energy of the solution *f* in Theorem 2.9 are bounded from below and above, uniformly in $t\in [0,T)$.

This immediately implies Theorem 1.1.

#### Proof of Theorem 1.1

By Theorem 2.9, there exist $T>0$ and a solution *f* of (2.3) such that $f\in W^{1,2}(0,T;L^2(I;{\mathbb {R}}^d))\cap L^2(0,T;W^{4,2}(I;{\mathbb {R}}^d))$. Consequently, *f* solves (1.3), since at the boundary we have

$$\begin{aligned} \partial _{s_f}f(t,y) = \frac{\partial _x f(t,y)}{|\partial _x f(t,y)|} = \frac{\gamma _0(y)\tau _y}{|\gamma _0(y)\tau _y|} = \tau _y \quad \text { for }t\in [0,T), y\in \partial I.&\end{aligned}$$

$$\square $$

#### Remark 2.11

Our assumption on the regularity of the initial datum is very natural. On the one hand, the space $W^{2,2}_{Imm}(I;{\mathbb {R}}^d)$ is the correct energy space associated to the elastic energy, so we would like to obtain short-time existence for an initial datum in $W^{2,2}_{Imm}(I;{\mathbb {R}}^d)$. In view of the linear problem in Theorem 2.1, working in the Sobolev scale one would hence need to pick $p\in (1,\infty )$ such that $W^{2,2}(I;{\mathbb {R}}^d)\hookrightarrow B^{4(1-\frac{1}{p})_{p,p}}(I;{\mathbb {R}}^d) ={\mathbb {Y}}_p^2$.

However, in order to estimate the denominator of the Lagrange multiplier $\lambda$, we want continuity of our solution with values in $W^{2,2}(I;{\mathbb {R}}^d)$. Using Proposition B.1 (i), this can be achieved if $B^{4(1-\frac{1}{p})_{p,p}}(I;{\mathbb {R}}^d) \hookrightarrow W^{2,2}(I;{\mathbb {R}}^d)$.

Clearly, this can only work for $p=2$. Moreover, for the same reason as above, even the introduction of time-weighted Sobolev spaces would not provide solutions with lower initial regularity.

#### Theorem 2.12

The solution $f \in {{\bar{B}}_{T,M}}$ in Theorem 2.9 is the unique solution of (2.3) in the whole space $W^{1,2}\left( 0,T; L^2(I;{\mathbb {R}}^d)\right) \cap L^2\left( 0,T; W^{4,2}(I;{\mathbb {R}}^d)\right)$.

#### Proof

First we note that any restriction of the solution $f \in {{\bar{B}}}_M$ to a smaller time interval $[0,{{\tilde{T}}}]$ is again the unique solution of (2.3) in ${{\bar{B}}}_M$ on $[0,{{\tilde{T}}}]$ by Theorem 2.9. Now, we let $T_1, T_2 > 0$ and assume that $f_i \in W^{1,2}\left( 0,T_i; L^2(I;{\mathbb {R}}^d)\right) \cap L^2\left( 0,T_i; W^{4,2}(I;{\mathbb {R}}^d)\right)$, $i = 1,2$ are two families of immersions satisfying (2.3) with $f_0 \in W^{2,2}_{{Imm}}(I)$. Without loss of generality we may assume that $T_1 \le T_2$. We claim that $f_2 |_{[0,T_1]} = f_1$.

To show the claim we define ${\bar{t}} = \sup \{t \in [0,T_1): f_1(s) = f_2(s) \, \forall \, 0 \le s \le t\}$. Note that ${\bar{t}}$ is well-defined by Proposition B.1 (i). We need to show that ${\bar{t}} = T_1$. To do so we first prove that ${\bar{t}} > 0$. Indeed, for $T \searrow 0$, we have $\Vert f_i|_{[0,T]} \Vert _{{\mathbb {X}}_T} \rightarrow 0$ by the dominated convergence theorem, and the same holds for the reference flow ${{\overline{f}}}$ from Definition 2.5. Thus, for $T > 0$ small enough, $f_i|_{[0,T]} \in {{\bar{B}}}_M$ for $i = 1,2$. Further decreasing $T>0$ if necessary we obtain from Theorem 2.9 that $f_1|_{[0,T]} = f_2|_{[0,T]}$ is the unique solution $f \in {{\bar{B}}}_M$. Thus, $f_1(s) = f(s) = f_2(s)$ for all $0 \le s \le T$, showing that ${\bar{t}} \ge T > 0$.

We now assume that ${\bar{t}} < T_1$. Since $f_i \in {\mathbb {X}}_{T_1} \hookrightarrow BUC([0,T_1],W^{2,2}(I;{\mathbb {R}}^d))$ and both solutions are immersed for all times, we find that $f_0 :=f_1({\bar{t}}) \in W^{2,2}_{Imm}(I;{\mathbb {R}}^d)$. Whence, by Theorem 2.9, there exist $M >0$, $T > 0$ such that (2.3) has a unique solution $f \in {{\bar{B}}}_M$. Observing that $f_i({\bar{t}} + t, \cdot )|_{0 \le t \le T_1-{\bar{t}}}$, $i = 1,2$ are both solutions to (2.3) with the same initial value $f_0$, we find by similar arguments as above that $f_1({\bar{t}} + \cdot ) = f = f_2({\bar{t}} + \cdot )$ on [0, *T*), contradicting the definition of ${\bar{t}}$. $\square$

## Parabolic smoothing

The goal of this section is to show that our solution *f* from Theorem 2.9 instantaneously becomes smooth.

### Theorem 3.1

Let $f_0\in W^{2,2}_{Imm}(I;{\mathbb {R}}^d)$ such that (1.4) and (1.7) are satisfied. Then, there exists $0<T_1\le T$ such that the solution *f* in Theorem 1.1 is smooth on $(0,T_1)$, i.e. $f\in {\mathcal {C}}^{\infty }((0,T_1)\times I;{\mathbb {R}}^d)$.

A close examination of the contraction estimates in Appendix C reveals that the critical embeddings are used, for instance in (C.12), (C.14) and (C.15). Thus, higher integrability of the nonlinearity cannot be obtained by standard estimates relying on Hölder’s inequality. Therefore, we cannot directly start the usual bootstrap argument to show smoothness. Instead, we use an instantaneous gain of regularity in the time variable, relying on Angenent’s parameter trick [2, 3], see also [18] and [44, Chapter 9]. Since we want to conclude smoothness in space *up to the boundary*, we first show increased regularity in time before deducing global smoothness up to the boundary by using parabolic Schauder theory. For the sake of readability, we omit the proof of the following proposition and refer to [46, Chapter 3, Section 3.1].

### Proposition 3.2

Let $f\in W^{1,2}(0,T;L^2(I;{\mathbb {R}}^d))\cap L^2(0,T;W^{4,2}(I;{\mathbb {R}}^d))$ be the unique solution of (2.3), given by Theorem 2.9. Then there exists $0<T_1<T$ such that $f\in {\mathcal {C}}^{\omega }((0,T_1); W^{2,2}(I,{\mathbb {R}}^d))$.

Next, we use the higher time regularity to improve the integrability of $\lambda$, which then allows us to start a bootstrap argument. First, we recall the following modification of [10, Lemma 4.3].

### Lemma 3.3

Let $f\in {\mathbb {X}}_{T,2}$ be a solution of (1.3). Then, we have

$$\begin{aligned}&|\lambda |\big (\ell - |p_1-p_0|\big )\le 2\ell \Vert \partial _t^{\perp }f \Vert _{L^1(\,\!\textrm{d}s)}+\int \nolimits _I|\vec {\kappa }|^2\,\!\textrm{d}s+\int \nolimits _I|\nabla _s\vec {\kappa }|\,\!\textrm{d}s. \end{aligned}$$

### Proof

We proceed as in [10, Lemma 4.3]. Let $l:[0,T)\times I\rightarrow {\mathbb {R}}^d$ be the parametrization of the line segment from $p_0$ to $p_1$ given by

$$\begin{aligned} l(t,x):=p_0 + \frac{\varphi (t,x)}{\ell }(p_1-p_0), \end{aligned}$$

with $\varphi (t, \xi ):=\int \nolimits _{0}^\xi |\partial _x f|\,\!\textrm{d}x$ for $(t,\xi )\in [0,T)\times I$. Then $l(t,0)=p_0$, $l(t,1)=p_1$ and $\partial _{s } l(t, \cdot ) = \frac{1}{\ell }(p_1-p_0)$. Therefore, using $\nabla _s^2\vec {\kappa }+ \frac{1}{2}|\vec {\kappa }|^2\vec {\kappa }= \nabla _s\left( \nabla _s\vec {\kappa }+ \frac{1}{2}|\vec {\kappa }|^2\partial _s f\right)$ (cf. [10, p. 1048]), we find after integrating by parts

$$\begin{aligned} \int \nolimits _{I} \langle \partial _t^{\perp } f, f-l\rangle \,\!\textrm{d}s&= \left\langle \left( \lambda -\frac{1}{2}|\vec {\kappa }|^2\right) \partial _s f - \nabla _s\vec {\kappa }, f-l \right\rangle \Bigg \vert _{\partial I} + \frac{1}{2} \int \nolimits _I|\vec {\kappa }|^2\,\!\textrm{d}s - \lambda \int \nolimits _I\,\!\textrm{d}s \\&\quad - \frac{1}{\ell } \int \nolimits _{I} \left\langle \nabla _s\vec {\kappa }+\frac{1}{2}|\vec {\kappa }|^2\partial _s f - \lambda \partial _s f, p_1-p_0\right\rangle \,\!\textrm{d}s . \end{aligned}$$

Consequently, since $f=l$ on the boundary, we have

$$\begin{aligned}&|\lambda |(\ell -|p_1-p_0|) = \left( 1-\frac{|p_1-p_0|}{\ell }\right) |\lambda |\int \nolimits _{I}\,\!\textrm{d}s \\&\quad \le \int \nolimits _{I} |\partial _t^{\perp }f|\,\!\textrm{d}s \Vert f-l \Vert _{\infty } + \frac{1}{2}\int \nolimits _{I}|\vec {\kappa }|^2\,\!\textrm{d}s + \frac{|p_1-p_0|}{2\ell }\int \nolimits _I|\vec {\kappa }|^2\,\!\textrm{d}s \\&\qquad + \frac{|p_1-p_0|}{\ell }\int \nolimits _I|\nabla _s\vec {\kappa }|\,\!\textrm{d}s. \end{aligned}$$

Using (1.7) and the simple estimate $\Vert f-l \Vert _{\infty } \le 2\ell$ yields the claim. $\square$

Note that a priori, the Lagrange multiplier $\lambda$ is only $L^2(0,T)$ for $f\in {\mathbb {X}}_{T,2}$. The next proposition improves this integrability, at least on a small timescale bounded away from zero.

### Lemma 3.4

Let *f* be the solution of (2.3) from Theorem 2.9 and let $T_1>0$ as in Proposition 3.2. Then, for any $0<\varepsilon <T_1$ we have $\lambda (f)\in L^4(\varepsilon ,T_1)$.

### Proof

As a consequence of Proposition 3.2, we have $\partial _t f\in {\mathcal {C}}^{\omega }((0,T_1);W^{2,2}(I;{\mathbb {R}}^d))$ and thus we get $\partial _t f\in {\mathcal {C}}^{0}([\varepsilon ,T_1]\times I;{\mathbb {R}}^d)$. Hence, Lemma 3.3 and (1.7) yield that $\lambda$ has the same integrability on $(\varepsilon , T_1)$ as $\int \nolimits _I |\nabla _s\vec {\kappa }|\,\!\textrm{d}s$. By Proposition A.1 (ii) and the uniform bounds on the arc-length element, cf. Remark 2.10, it suffices to show $\partial _x^3 f \in L^4(\varepsilon ,T_1;L^1(I;{\mathbb {R}}^d))$, since $\partial _x^2f\in {\mathcal {C}}^{0}([\varepsilon ,T_1];L^2(I;{\mathbb {R}}^d))$. In fact using Proposition B.3 (i) as in (C.12), we even get $\partial _x^3 f\in L^4(\varepsilon , T_1; L^2(I;{\mathbb {R}}^d))$. $\square$

The improved integrability of $\lambda$ in Lemma 3.4 enables us to start a bootstrap argument to increase the Sobolev regularity of our solution in Theorem 2.9. Note that by Sobolev embeddings, in order to prove smoothness of our solution it suffices to reach ${\mathbb {X}}_{T,p}$ with $p>5$, see Lemma 3.6.

### Lemma 3.5

Let *f* be as in Theorem 2.9, let $T_1>0$ be as in Proposition 3.2 and let $0<\varepsilon <T_1$. Then $f\in W^{1,20}(\varepsilon , T_1; L^{20}(I;{\mathbb {R}}^d))\cap L^{20}(\varepsilon ,T_1;W^{4,20}(I;{\mathbb {R}}^d))$.

### Proof

See [46, Chapter 3, Section 3.3]. $\square$

Finally, Theorem 3.1 follows from parabolic Schauder theory and the following

### Lemma 3.6

Let *f* be the solution of (2.3) constructed in Theorem 2.9. If there exist $p>5$ and $\varepsilon >0$ such that $f \in W^{1,p}\left( \varepsilon ,T_1; L^p(I;{\mathbb {R}}^d)\right) \cap L^p\left( \varepsilon ,T_1; W^{4,p}(I;{\mathbb {R}}^d)\right)$ then $f \in {\mathcal {C}}^\infty ((\varepsilon , T_1) \times I;{\mathbb {R}}^d)$.

### Proof

See [46, Chapter 3, Section 3.4]. $\square$

Now, Theorem 3.1 is immediate.

### Proof of Theorem 3.1

The solution *f* in Theorem 1.1 is exactly the solution *f* in Theorem 2.9. By Lemma 3.5 we have $f\in W^{1,20}(\varepsilon ,T_1;L^{20}(I;{\mathbb {R}}^d))\cap L^{20}(\varepsilon ,T_1;L^{20}(I;{\mathbb {R}}^d))$ for any $0<\varepsilon <T_1$. Hence, by Lemma 3.6, we find that $f\in {\mathcal {C}}^{\infty }((\varepsilon ,T_1)\times I;{\mathbb {R}}^d))$. $\square$

## Long-time behaviour and the proof of Theorem 1.2

In this section, we use the long-time existence result in [10] to show the existence of a global solution of (1.3). Moreover, we prove and use a *refined Łojasiewicz–Simon gradient inequality* to conclude convergence after reparametrization.

### Long-time existence after reparametrization

As a first step towards proving Theorem 1.2, we establish long-time existence and subconvergence after reparametrization for our solution. The key ingredient is the smoothness of our solution and [10, Theorem 1.1].

#### Theorem 4.1

Let $f\in W^{1,2}(0,T;L^2(I;{\mathbb {R}}^d))\cap L^2(0,T;W^{4,2}(I;{\mathbb {R}}^d))$ be as in Theorem 2.9 and let $0<\varepsilon <T$. Then, there exist ${\bar{\varepsilon }}\in (\varepsilon , T)$ and ${\hat{f}}\in {\mathcal {C}}^{\infty }((0,\infty )\times I;{\mathbb {R}}^d)$ satisfying (1.3) such that

(i) $${\hat{f}}(t,x) = f(t,x)$$ for all $$0\le t\le \varepsilon , x\in I$$;
(ii) ${\hat{f}}(t, \cdot )$ has zero tangential velocity for all $t\ge {\bar{\varepsilon }}$;
(iii) ${\hat{f}}$ subconverges smoothly as $t\rightarrow \infty$, after reparametrization with constant speed, to a constrained elastica, i.e. a solution (1.8).

#### Proof

By Theorem 3.1, the solution *f* in Theorem 2.9 is instantaneously smooth. Thus, to simplify notation we may assume $f\in {\mathcal {C}}^{\infty }([\varepsilon , T]\times I;{\mathbb {R}}^d)$ for some $\varepsilon >0$ after possibly reducing $T>0$. Moreover, we may also assume a uniform bound from below on the arc-length element using Remark 2.10.

Let $\theta :=\langle \partial _t {f}, \partial _{s_{{f}}} {f}\rangle$ be the tangential velocity of *f*. By the smoothness of *f* and the bound on the arc-length element, the function $(t,r)\mapsto \frac{\theta (t,r)}{|\partial _x f(t,r)|}$ is globally Lipschitz continuous on $[\varepsilon , T]\times I$. For each $x\in I$, we consider the initial value problem

4.1 $$\begin{aligned} \left\{ \begin{array}{llr} \partial _t \Phi (t,x) &{}= - \frac{\theta (t,\Phi (t,x))}{|\partial _x {f}(t, \Phi (t,x))|} \\ \Phi (\varepsilon ,x) &{}= x. \end{array}\right. \end{aligned}$$

By classical ODE theory, there exist $\varepsilon < {\hat{T}}\le T$ and a smooth family of reparametrizations $\Phi :[\varepsilon ,{\hat{T}}]\times I\rightarrow I$ satisfying (4.1) and

4.2 $$\begin{aligned} \Phi (t,y)&= y \quad \text { for }t\in [\varepsilon ,{\hat{T}}], y\in \partial I \nonumber \\ \partial _x \Phi (t,x)&>0 \quad \text { for all }(t,x)\in [\varepsilon ,{\hat{T}}]\times I. \end{aligned}$$

Therefore, $\Phi (t, \cdot )$ is strictly increasing and a diffeomorphism of *I* for each $t\in [\varepsilon , {\hat{T}}]$. A direct computation yields that the reparametrization ${f}_1(t,x):={f}(t, \Phi (t,x))$ satisfies

$$\begin{aligned} \partial _t {f}_1(t,x)&= \partial _t {f}(t,\Phi (t,x)) + \partial _x{f}(t, \Phi (t,x))\partial _t \Phi (t,x) \\&= \partial _t^{\perp }{f}(t, \Phi (t,x)) + \theta (t, \Phi (t,x)) \partial _{s_{{f}}}{f}(t, \Phi (t,x)) \\ {}&\quad + \partial _x {f}(t, \Phi (t,x))\partial _t \Phi (t,x) \\&=\partial _t^{\perp }{f}(t, \Phi (t,x)) \\&= -\nabla _{s_{f_1}}^2\vec {\kappa }_{f_1}(t,x) -\frac{1}{2}|\vec {\kappa }_{f_1}(t,x)|^2\vec {\kappa }_{f_1}(t,x) + \lambda ({f_1})(t)\vec {\kappa }_{f_1}(t,x), \end{aligned}$$

using that *f* solves (1.3) and the transformation of the geometric quantities. For the boundary conditions, let $t\in [\varepsilon ,{\hat{T}}]$, $y\in \partial I$ and note that $f_1(t,y)=f(t,y)=p_y$ and $\partial _{s_{f_1}} f_1(t,y) =\partial _{s_f}f(t,y) =\tau _y$ by (4.2). Consequently, $f_1$ is a smooth solution of (1.3) on $[\varepsilon , {\hat{T}}]$ with tangential velocity zero and smooth initial datum $f(\varepsilon )$. By [10, Theorem 1.1], $f_1$ can be extended to a global smooth solution ${\bar{f}}$ on $[\varepsilon , \infty )$ which subconverges, after reparametrization with constant speed, to a constrained elastica as $t\rightarrow \infty$.

In particular, we have the identity

4.3 $$\begin{aligned} {\bar{f}}(t,x) = f(t,\Phi (t,x))\quad \text { for all }\varepsilon \le t\le {\hat{T}}. \end{aligned}$$

Now, let $\varepsilon<{\bar{\varepsilon }}<{\hat{T}}$ and $\Psi :[0, {\hat{T}}]\times I \rightarrow I$ be a smooth family of reparametrizations with

4.4 $$\begin{aligned} \Psi (t,x) = x\quad \text { for all }0\le t\le \varepsilon ; \qquad \Psi (t,x) =\Phi (t,x) \quad \text { for all }{\bar{\varepsilon }}\le t\le {\hat{T}}. \end{aligned}$$

The existence of such a $\Psi$ is proven in Lemma D.1. We now define

$$\begin{aligned} {\hat{f}}(t,x) :=\left\{ \begin{array}{ll} f(t,\Psi (t,x)) &{} \text { for }0\le t\le {\hat{T}}, x\in I\\ {\bar{f}}(t,x) &{} \text { for } t\ge {\bar{\varepsilon }}, x\in I. \end{array}\right. \end{aligned}$$

Note that ${\hat{f}}$ is clearly smooth in *x* for every $t\ge 0$ fixed. It is also smooth in *t* for fixed $x\in I$, by (4.3) and (4.4). Property (i) follows from (4.4). Furthermore, by definition of ${\hat{f}}$ on $[{\bar{\varepsilon }},\infty )\times I$ we find that ${\hat{f}}={\bar{f}}$ has zero tangential velocity and hence (ii) is satisfied. The last property follows since the asymptotic behaviour of ${\hat{f}}$ is inherited from ${\bar{f}}$. $\square$

### The length-preserving elastic flow as a gradient flow on a Hilbert manifold

In this section, we show that the flow (1.3) is in fact a gradient flow on a suitable submanifold of curves.

#### Proposition 4.2

Let $p_0,p_1 \in {\mathbb {R}}^d, \tau _0, \tau _1 \in {\mathbb {S}}^{d-1}$ and $\ell \in {\mathbb {R}}$ such that (1.7) holds. Then

$$\begin{aligned} {\mathcal {X}} :=\left\{ f\in W^{4,2}_{Imm}(I;{\mathbb {R}}^d)\mid f(y)=p_y \text { and }\partial _s f(y) = \tau _y \text { for } y\in \partial I, {\mathcal {L}}(f) = \ell \right\} . \end{aligned}$$

is a weak Riemannian splitting analytic submanifold of $W^{4,2}(I;{\mathbb {R}}^d)$ with codimension $4d-1$.

#### Proof

By the Sobolev embedding $W^{4,2}(I;{\mathbb {R}}^d)\hookrightarrow {\mathcal {C}}^{1}(I;{\mathbb {R}}^{d})$, the set of $W^{4,2}$-immersions denoted by $W^{4,2}_{Imm}(I;{\mathbb {R}}^d)$ is open in $W^{4,2}(I;{\mathbb {R}}^{d})$. The function

$$\begin{aligned} {\mathcal {G}}:W^{4,2}_{Imm}(I;{\mathbb {R}}^{d}) \rightarrow {\mathbb {R}}\times ({\mathbb {R}}^d)^2\times ({\mathbb {S}}^{d-1})^2, {\mathcal {G}}(f) :=\begin{pmatrix} {\mathcal {L}}(f) \\ f(0) \\ f(1) \\ \partial _{s_f}f(0) \\ \partial _{s_f}f(1) \end{pmatrix} \end{aligned}$$

is an analytic map. Moreover, its differential is given by

$$\begin{aligned}&d {\mathcal {G}}_f :W^{4,2}(I;{\mathbb {R}}^d)\rightarrow {\mathbb {R}}\times ({\mathbb {R}}^d)^2 \times {\mathcal {T}}_{\partial _{s}f(0)}{\mathbb {S}}^{d-1}\times {\mathcal {T}}_{\partial _{s}f(1)}{\mathbb {S}}^{d-1},\\&d {\mathcal {G}}_f(u) = \begin{pmatrix} -\int \nolimits _{I} \langle \vec {\kappa }_{{f}}, u\rangle \,\!\textrm{d}s_{f} \\ u(0) \\ u(1) \\ \frac{\partial _x u(0)}{|\partial _x f(0)|} - \frac{\langle \partial _x u(0), \partial _x f(0)\rangle \partial _x f(0)}{|\partial _x f(0)|^3} \\ \frac{\partial _x u(1)}{|\partial _x f(1)|} - \frac{\langle \partial _x u(1), \partial _x f(1)\rangle \partial _x f(1)}{|\partial _x f(1)|^3} \end{pmatrix} \end{aligned}$$

for $f\in W^{4,2}_{Imm}(I;{\mathbb {R}}^{d})$ and $u \in W^{4,2}(I;{\mathbb {R}}^{d})$. It is not difficult to see that $d{\mathcal {G}}_f$ is surjective if $f\in {\mathcal {X}} = {\mathcal {G}}^{-1}\left( \{(\ell , p_0, p_1, \tau _0, \tau _1)^T\}\right)$. Indeed, let $\alpha \in {\mathbb {R}}$, $q_y\in {\mathbb {R}}^{d}$, $z_y \in T_{\partial _{s}f(y)}{\mathbb {S}}^{d-1}$ for $y=0,1$. We have ${ {\mathcal {T}}_{\partial _s f(y)}{\mathbb {S}}^{d-1} = \{z\in {\mathbb {R}}^{d} \mid \langle z, \partial _x f(y)\rangle =0 \}}$. Clearly, we can find an immersed curve $u\in W^{4,2}(I;{\mathbb {R}}^{d})$ with $u(y)=q_y$ and $\frac{\partial _x u(y)}{|\partial _x f(y)|} = z_y$ for $y=0,1$. Now, using the characterization of the tangent space, for $v\in {\mathcal {C}}^{\infty }_0(I;{\mathbb {R}}^d)$ we find

$$\begin{aligned} d{\mathcal {G}}_f(u+v) = \begin{pmatrix} -\int \nolimits _{I} \langle \vec {\kappa }_{{f}}, u+v\rangle \,\!\textrm{d}s_{f} \\ q_0\\ q_1\\ z_0\\ z_1\\ \end{pmatrix}, \end{aligned}$$

since adding *v* does not change the boundary behaviour. Moreover, as $\vec {\kappa }_{f}\not \equiv 0$ using $f\in {\mathcal {X}}$ and (1.7), we can choose *v* such that $\int \nolimits _{I}\langle \vec {\kappa }_f, v\rangle \,\!\textrm{d}s_{f} =\varepsilon \ne 0$. Setting $\beta :=\int \nolimits _{I}\langle \vec {\kappa }_f, u\rangle \,\!\textrm{d}s_{f}$ and $w:=u -\frac{\alpha +\beta }{\delta } v$, we find $\int \nolimits _{I} \langle \vec {\kappa }_f, w\rangle \,\!\textrm{d}s_{f} = \beta - (\alpha + \beta ) = -\alpha$; hence, we have shown $d{\mathcal {G}}_f(w) = (\alpha , q_0, q_1, z_0, z_1)$, so $d{\mathcal {G}}_f$ is surjective.

Consequently, ${\mathcal {X}}\subset W^{4,2}(I;{\mathbb {R}}^{d})$ is a splitting submanifold by [1, Theorem 3.5.4] with codimension $1+2d+2(d-1)=4d-1$. Like in [45], the analytic form of the implicit function theorem can be used to show that ${\mathcal {X}}$ is in fact analytic. The tangent space is given by

4.5 $$\begin{aligned} {\mathcal {T}}_f {\mathcal {X}}&= \ker d{\mathcal {G}}_f \nonumber \\&= \left\{ u \in W^{4,2}(I;{\mathbb {R}}^{d})\mid u=0 \text { on }\partial I, \partial _x^{\perp _f}u = 0\text { on }\partial I, \int \nolimits _{I}\langle \vec {\kappa }_f, u\rangle \,\!\textrm{d}s_{f}=0\right\} . \end{aligned}$$

Since (1.3) is a $L^2(\,\!\textrm{d}s_f)$ gradient flow, it is natural to endow ${\mathcal {X}}$ with the Riemannian metric $\langle u,v\rangle _{L^2(\,\!\textrm{d}s_f)} = \int \nolimits _{I} \langle u,v\rangle \,\!\textrm{d}s_{f}$ for $u,v \in {\mathcal {T}}_f {\mathcal {X}}$. Note that since ${\mathcal {T}}_f{\mathcal {X}}$ is certainly not complete with respect to the induced norm, the metric is only *weakly Riemannian* (cf. [1, Definition 5.2.12]). $\square$

It is not difficult to see that by (4.5) the right-hand side of the evolution (1.1) is the projection of the full $L^2 (\,\!\textrm{d}s_f)$-gradient $\nabla {\mathcal {E}}(f)$ onto the $L^2(\,\!\textrm{d}s_f)$-closure of the tangent space ${\mathcal {T}}_f{\mathcal {X}}$. This implies that (1.1) is the gradient flow of ${\mathcal {E}}$ on the manifold ${\mathcal {X}}$.

### The constrained Łojasiewicz–Simon gradient inequality

In this subsection, we establish a Łojasiewicz–Simon inequality for ${\mathcal {E}}$ on ${\mathcal {X}}$. To do so, we have to deal with the invariance of both energies ${\mathcal {E}}$ and ${\mathcal {G}}$, which unfortunately creates large kernels for their first and second variations. Like in [8, 13], we work around this issue by restricting the energy to normal directions and using the implicit function theorem.

In the following, we always assume that the assumptions (1.4) and (1.7) are satisfied.

#### Definition 4.3

Fix ${\bar{f}}\in {\mathcal {X}}$ and define $V_c:=W^{4,2}(I;{\mathbb {R}}^d)\cap W^{2,2}_{0}(I;{\mathbb {R}}^d)$. We define the *space of normal vector fields along* ${\bar{f}}$ by

$$\begin{aligned} W^{4,2,\perp }(I;{\mathbb {R}}^d) :=\{ f\in W^{4,2}(I;{\mathbb {R}}^d) \mid \langle f, \partial _x {\bar{f}}\rangle = 0 \text { on } I\}. \end{aligned}$$

Moreover, we define $H^{\perp }:=L^{2,\perp }(I;{\mathbb {R}}^d):=\{ u\in L^{2}(I;{\mathbb {R}}^d)\mid \langle u, \partial _x {\bar{f}} \rangle = 0 \text { a.e.}\}$ and $V_c^{\perp } :=V_c \cap W^{4,2,\perp }(I;{\mathbb {R}}^d)$. Both are Hilbert spaces and the $L^2$-orthogonal projection onto $H^{\perp }$ is given by the pointwise projection $P^{\perp }(f) :=f - \langle f, \partial _s {\bar{f}}\rangle \partial _s {\bar{f}}$.

Moreover, by the embedding $W^{4,2}(I;{\mathbb {R}}^d)\hookrightarrow {\mathcal {C}}^{1}(I;{\mathbb {R}}^d)$ there exists $\varepsilon >0$ small enough such that for all $u\in W^{4,2,\perp }(I;{\mathbb {R}}^d)$ with $\Vert u \Vert _{W^{4,2}}<\varepsilon$, the curve $f={\bar{f}}+u$ is immersed. Defining $U_\varepsilon :=\{ u\in V_c^{\perp } \mid \Vert u \Vert _{W^{4,2}}<\varepsilon \}$ we consider the energies

$$\begin{aligned}&L :U_\varepsilon \rightarrow {\mathbb {R}}, \quad L(u)={\mathcal {L}}({\bar{f}}+u) \text { and }\\&E:U_\varepsilon \rightarrow {\mathbb {R}}, \quad E(u)={\mathcal {E}}({\bar{f}}+u). \end{aligned}$$

We have the following result.

#### Proposition 4.4

(cf. [13, Proof of Theorem 3.1, Remark 3.3]) The energy *E* satisfies the following properties.

1. $$E:U_\varepsilon \rightarrow {\mathbb {R}}$$ is analytic;
2. its gradient $\nabla E :U_\varepsilon \rightarrow H^{\perp }$ is analytic;
3. the derivative $(\nabla E)^{\prime }(0):V^{\perp }_{c} \rightarrow H^{\perp }$ is Fredholm with index zero.

It is well known that this is sufficient to prove a Łojasiewicz–Simon gradient inequality for *E* (cf. [7, Corollary 3.11]), [13, Theorem 3.1], [45, Theorem 1.2], [43, Corollary 2.6]). However, in order to conclude a *constrained* or *refined* Łojasiewicz–Simon gradient inequality, cf. (16) in [45], we also need to analyse the length functional.

#### Proposition 4.5

The energy *L* satisfies the following properties.

1. $$L:U_\varepsilon \rightarrow {\mathbb {R}}$$ is analytic.
2. The gradient map $\nabla L :U_\varepsilon \rightarrow H^{\perp }$ is analytic.
3. The derivative $(\nabla L)^{\prime }(0):V_{c}^{\perp }\rightarrow H^{\perp }$ is compact.
4. $$L(0)=\ell $$ and $$\nabla L(0)\ne 0$$.

#### Proof

 

1. The map $U_{\varepsilon }\rightarrow {\mathcal {C}}(I;{\mathbb {R}}^d), u \mapsto |\partial _x({\bar{f}}+u)|$ is analytic by [13, Lemma 3.4, 1.], and hence so is *L*.
2. The $H^{\perp }$-gradient of *L* is given by $\nabla L(u) = -P^{\perp } \left( \vec {\kappa }_{{\bar{f}}+u}|\partial _{x}({\bar{f}}+u)|\right)$. Note that the map $U_{\varepsilon }\rightarrow L^{2}(I;{\mathbb {R}}^d), u \mapsto \vec {\kappa }_{{\bar{f}}+u}$ is analytic by [13, Lemma 3.4, 3.]. Since the multiplication $L^2(I;{\mathbb {R}}^d)\times L^{\infty }(I;{\mathbb {R}}) \rightarrow L^2(I;{\mathbb {R}}^d), (f,\phi )\mapsto f\phi$ is analytic, so is the map $U_{\varepsilon }\rightarrow L^2(I;{\mathbb {R}}^d), u\mapsto \vec {\kappa }_{{\bar{f}}+u}|\partial _{x}({\bar{f}}+u)|$. The continuity and linearity of $P^{\perp }:L^2(I;{\mathbb {R}}^d)\rightarrow H^{\perp }$ yield the claim.
3. We compute the second derivative using standard formulas for the variation of geometric quantities (see for instance [17, Lemma 2.1]). We have
  $$\begin{aligned} (\nabla L)^{\prime }(0) u&= \left. \frac{\!\textrm{d}}{\!\textrm{d}t}\right| _{t=0}\nabla L(tu) = -\left. \frac{\!\textrm{d}}{\!\textrm{d}t}\right| _{t=0}P^{\perp } \left( \vec {\kappa }_{{\bar{f}}+u}|\partial _{x}({\bar{f}}+u)|\right) \\&= -P^{\perp } \left. \frac{\!\textrm{d}}{\!\textrm{d}t}\right| _{t=0}\vec {\kappa }_{{\bar{f}}+tu} |\partial _{x}{\bar{f}}| - P^{\perp } \vec {\kappa }_{{\bar{f}}} \left. \frac{\!\textrm{d}}{\!\textrm{d}t}\right| _{t=0}|\partial _x ({\bar{f}}+tu)|\\&= -\left( \nabla _{s_{{\bar{f}}}}^2 u + \langle u, \vec {\kappa }_{{\bar{f}}}\rangle \vec {\kappa }_{{\bar{f}}}\right) |\partial _{x}{\bar{f}}| + \vec {\kappa }_{{\bar{f}}}\langle u, \vec {\kappa }_{{\bar{f}}}\rangle |\partial _x {\bar{f}}|. \end{aligned}$$
  In particular, the operator $(\nabla L)^{\prime }(0):V_c^{\perp }\rightarrow H^{\perp }$ is only of second order in *u*, hence compact by the Rellich–Kondrachov Theorem [23, Theorem 7.26].
4. $L(0)={\mathcal {L}}({\bar{f}})=\ell$ since ${\bar{f}}\in {\mathcal {X}}$. Since we have $|{\bar{f}}(1)-{\bar{f}}(0)| = |p_1-p_0| < \ell$, ${\bar{f}}$ cannot be part of a straight line; hence, $\vec {\kappa }_{{\bar{f}}}\not \equiv 0$ and also $|\partial _x {\bar{f}}|\ne 0$ since ${\bar{f}}$ is immersed.

$$\square $$

This enables us to conclude the inequality in normal directions.

#### Theorem 4.6

Suppose ${\bar{f}}\in {\mathcal {X}}$ is a constrained elastica. Then, there exist $C, \sigma >0$ and $\theta \in (0,\frac{1}{2}]$ such that for all $f={\bar{f}}+u\in {\mathcal {X}}$ with $u\in V_c^{\perp }$ and $\Vert u \Vert _{W^{4,2}}\le \sigma$ we have

$$\begin{aligned} |{\mathcal {E}}(f)-{\mathcal {E}}({\bar{f}})|^{1-\theta }\le C\Vert \nabla _{L^2(\,\!\textrm{d}s_{f})} {\mathcal {E}}(f) + \lambda (f)\nabla _{L^2(\,\!\textrm{d}s_{f})} {\mathcal {L}}(f) \Vert _{L^2(\,\!\textrm{d}s_{f})}. \end{aligned}$$

#### Proof

First, we verify the conditions of [45, Corollary 5.2] for the energy *E* and the constraint ${\mathcal {G}}(u)=L(u)-\ell$ on the spaces $V=V_{c}^{\perp }, H=H^{\perp }$. Note that $\nabla {\mathcal {G}} = \nabla {L}$. Clearly, $V_c^{\perp }\hookrightarrow H^{\perp }$ densely. Assumptions (ii) and (iii) follow from Proposition 4.4, whereas assumptions (iv)–(-vi) are satisfied by Proposition 4.5. Note that $u=0$ is a constrained critical point of *E* on ${\mathcal {M}}={\mathcal {G}}^{-1}(\{0\})$ since ${\bar{f}}$ is a constrained elastica.

Then, by [45, Corollary 5.2] $E\vert _{{\mathcal {M}}}$ satisfies a constrained Łojasiewicz–Simon gradient inequality, i.e. there exist $C, \sigma >0$ and $\theta \in (0,\frac{1}{2}]$ such that for all $u\in {\mathcal {M}}$ with $\Vert u \Vert _{W^{4,2}}\le \sigma$ we have

$$\begin{aligned} |E(u)-E(0)|^{1-\theta }\le C \Vert P(u)\nabla E(u) \Vert _{L^2}, \end{aligned}$$

where $P(u):H^{\perp }\rightarrow H^{\perp }$ denotes the orthogonal projection onto the closure of the tangent space $\overline{T_u {\mathcal {M}}} = \{ y\in H^{\perp }\mid \langle \nabla L(u), y\rangle _{L^2} = 0\}$ (cf. [45, Proposition 3.3]). Therefore, for

$$\begin{aligned} {\lambda }(f) = \frac{\langle \vec {\kappa }_f, \nabla {\mathcal {E}}(f)\rangle _{L^2(\,\!\textrm{d}s_{f})}}{\Vert \vec {\kappa }_f \Vert _{L^2(\,\!\textrm{d}s_{f})}^2} \end{aligned}$$

as in (1.2) with $f={\bar{f}}+u$ we have the estimate

$$\begin{aligned} \Vert P(u)\nabla E(u) \Vert _{L^2} = \Vert P(u)\left( \nabla E(u) + {\lambda } \nabla L(u)\right) \Vert _{L^2} \le \Vert \nabla E(u) + {\lambda } \nabla L(u) \Vert _{L^2}. \end{aligned}$$

Moreover, we have $\nabla E(u) = \nabla _{L^2(\,\!\textrm{d}s_{f})} {\mathcal {E}}(f) |\partial _x f|$ and $\nabla L(u) = \nabla _{L^2(\,\!\textrm{d}s_{f})} {\mathcal {L}}(f) |\partial _x f|$. Consequently,

$$\begin{aligned} \Vert P(u)\nabla E(u) \Vert _{L^2}&\le \Vert P(u)\left( \nabla E(u) + {\lambda } \nabla L(u)\right) \Vert _{L^2} \\ {}&\le \Vert \nabla _{L^2(\,\!\text {d}s_{f})} {\mathcal {E}}(f) |\partial _x f| + {\lambda } \nabla _{L^2(\,\!\text {d}s_{f})} {\mathcal {L}}(f) |\partial _x f| \Vert _{L^2}\\ {}&\le \Vert \partial _x f \Vert _{L^{\infty }}^{\frac{1}{2}}\Vert \nabla _{L^2(\,\!\text {d}s_{f})} {\mathcal {E}}(f) + {\lambda } \nabla _{L^2(\,\!\text {d}s_{f})} {\mathcal {L}}(f) \Vert _{L^2(\,\!\text {d}s_{f})}. \end{aligned}$$

Reducing $\sigma >0$ if necessary, we may assume that $\Vert \partial _x f \Vert _{L^{\infty }}$ is uniformly bounded for $\Vert f-{\bar{f}} \Vert _{W^{4,2}}\le \sigma$ by the Sobolev embedding theorem. This proves the claim. $\square$

We use this to prove the full constrained Łojasiewicz–Simon gradient inequality for not necessarily normal variations via the following reparametrization argument.

#### Lemma 4.7

([13, Lemma 4.1]) Let ${\bar{f}}\in W^{5,2}(I;{\mathbb {R}}^d)$ be a regular curve. Then, there exists $\sigma >0$ such that for all $\psi \in V_c$ with $\Vert \psi \Vert _{W^{4,2}}\le \sigma$, there exists a $W^{4,2}$-diffeomorphism $\Phi :I\rightarrow I$ such that

4.6 $$\begin{aligned} ({\bar{f}}+\psi )\circ \Phi = {\bar{f}}+\eta \end{aligned}$$

for some $\eta \in V_c^{\perp }$. Moreover, given $\sigma >0$ there exists ${\tilde{\sigma }}={\tilde{\sigma }}({\bar{f}}, \sigma )>0$ such that for all $\psi \in V_c$ with $\Vert \psi \Vert _{W^{4,2}}\le {\tilde{\sigma }}$ we have the above representation with $\Vert \eta \Vert _{W^{4,2}}\le \sigma$.

#### Theorem 4.8

Let ${\bar{f}}\in {\mathcal {X}}\cap W^{5,2}(I;{\mathbb {R}}^d)$ be a constrained elastica. Then there exist $C, \sigma >0$ and $\theta \in (0,\frac{1}{2}]$ such that

$$\begin{aligned} |{\mathcal {E}}({f})-{\mathcal {E}}({\bar{f}})| \le C \Vert \nabla _{L^2(\,\!\text {d}s_{f})} {\mathcal {E}}(f) + \lambda (f)\nabla _{L^2(\,\!\text {d}s_{f})} {\mathcal {L}}(f) \Vert _{L^2(\,\!\text {d}s_{f})}, \end{aligned}$$

for all $f\in {\mathcal {X}}$ such that $\Vert f-{\bar{f}} \Vert _{W^{4,2}}\le \sigma$.

#### Proof

Let $C, \sigma >0, \theta \in (0,\frac{1}{2}]$ as in Theorem 4.6, ${\bar{f}}\in {\mathcal {X}}$ be a constrained critical point of ${\mathcal {E}}$ on ${\mathcal {X}}$. By the regularity assumption on ${\bar{f}}$, we may use Lemma 4.7.

Thus, we find ${\tilde{\sigma }}>0$ such that (4.6) holds for all $\psi \in V_c$ with $\Vert \psi \Vert _{W^{4,2}}\le {\tilde{\sigma }}$ for some $\eta \in V_c^{\perp }$ with $\Vert \eta \Vert _{W^{4,2}}\le \sigma$. Let $f\in {\mathcal {X}}$ such that $\Vert f-{\bar{f}} \Vert _{W^{4,2}}\le {\tilde{\sigma }}$. Then by Lemma 4.7, there exist a diffeomorphism $\Phi :I \rightarrow I$ and $\eta \in V_c^{\perp }$ such that $f\circ \Phi = {\bar{f}}+\eta$.

Note that with $f, {\bar{f}}\in {\mathcal {X}}$ we also get $f\circ \Phi = {\bar{f}}+\eta \in {\mathcal {X}}$, since ${\mathcal {L}}(f) = {\mathcal {L}}(f\circ \Phi ) = \ell$. Since the elastic energy is invariant under reparametrization, we hence get using Theorem 4.6

4.7 $$\begin{aligned}&\left| {\mathcal {E}}(f)-{\mathcal {E}}({\bar{f}})\right| ^{1-\theta } =\left| {\mathcal {E}}({\bar{f}}+\eta )-{\mathcal {E}}({\bar{f}})\right| ^{1-\theta } \nonumber \\ {}&\quad \le C\Vert \nabla _{L^2(\,\!\text {d}s_{{\bar{f}}+\eta })} {\mathcal {E}}({\bar{f}}+\eta ) + \lambda ({\bar{f}}+\eta )\nabla _{L^2(\,\!\text {d}s_{{\bar{f}}+\eta })} {\mathcal {L}}({\bar{f}}+\eta ) \Vert _{L^2(\,\!\text {d}s_{{\bar{f}}+\eta })}.\end{aligned}$$

Since $\lambda$ and the gradients are geometric, i.e. transform correctly under reparametrizations, we have

$$\begin{aligned} \lambda ({\bar{f}}+\eta )&= \lambda (f\circ \Phi ) = \lambda (f), \\ \nabla _{L^2(\,\!\text {d}s_{{\bar{f}}+\eta })} {\mathcal {E}}({\bar{f}}+\eta )&= \nabla _{L^2(\,\!\text {d}s_{f})} {\mathcal {E}}(f) \circ \Phi \\ \nabla _{L^2(\,\!\text {d}s_{{\bar{f}}+\eta })} {\mathcal {L}}({\bar{f}}+\eta )&= \nabla _{L^2(\,\!\text {d}s_{f})} {\mathcal {L}}(f) \circ \Phi . \end{aligned}$$

Consequently, we obtain

$$\begin{aligned}&\Vert \nabla _{L^2(\,\!\text {d}s_{{\bar{f}}+\eta })} {\mathcal {E}}({\bar{f}}+\eta ) + \lambda ({\bar{f}}+\eta )\nabla _{L^2(\,\!\text {d}s_{{\bar{f}}+\eta })} {\mathcal {L}}({\bar{f}}+\eta ) \Vert _{L^2(\,\!\text {d}s_{{\bar{f}}+\eta })} \\ {}&\quad = \Vert \nabla _{L^2(\,\!\text {d}s_{f})} {\mathcal {E}}(f)\circ \Phi + \lambda (f)\nabla _{L^2(\,\!\text {d}s_{f})} {\mathcal {L}}(f)\circ \Phi \Vert _{L^2(\,\!\text {d}s_{f\circ \Phi })} \\ {}&\quad = \Vert \nabla _{L^2(\,\!\text {d}s_{f})} {\mathcal {E}}(f) + \lambda (f)\nabla _{L^2(\,\!\text {d}s_{f})} {\mathcal {L}}(f) \Vert _{L^2(\,\!\text {d}s_{f})}. \end{aligned}$$

Together with (4.7), this implies the Łojasiewicz–Simon gradient inequality for the elastic energy on ${\mathcal {X}}$. $\square$

### Convergence

In previous works (see e.g. [8, p. 358 – 359] and [13, p. 2188 – 2191]), a lot of PDE theory and a priori parabolic Schauder estimates are needed to apply the Łojasiewicz–Simon gradient inequality to conclude convergence for geometric problems. In this section, we introduce a novel inequality (see Lemma 4.10) which enables us to significantly shorten this lengthy argument in the proof of Theorem 1.2. We exploit the explicit structure of the constant speed reparametrization and the length bound to control the full velocity of the constant speed parametrization by the purely normal velocity of the original evolution.

#### Definition 4.9

Let $T\in (0, \infty ]$ and let $f:[0,T)\times I\rightarrow {\mathbb {R}}^d$ be a family of immersed curves in ${\mathbb {R}}^d$. The *constant speed* ${\mathcal {L}}(f(t))$ *reparametrization* ${\tilde{f}}(t)$ *of* *f*(*t*) is given by ${\tilde{f}}(t,x):=f(t, \psi (t,x))$ where $\psi (t, \cdot ):{I}\rightarrow {I}$ is the inverse of $\varphi (t,\cdot ):{I}\rightarrow {I}$ given by

$$\begin{aligned} \varphi (t,x) := \frac{1}{{\mathcal {L}}(f(t))}\int \nolimits _0^x |\partial _x f(t,z)|\,\!\textrm{d}z = \frac{1}{{\mathcal {L}}(f(t))}\int \nolimits _0^x\, \!\textrm{d}s_{f(t)}. \end{aligned}$$

#### Lemma 4.10

Suppose $T\in (0, \infty ]$ and $f:[0,T)\times I\rightarrow {\mathbb {R}}^d$ is a family of curves in ${\mathbb {R}}^d$, such that $f(t,0)=p_0, f(t,1)=p_1$ and ${\mathcal {L}}(f(t)) >0$ for all $t\in (0,T]$. Then, if ${\tilde{f}}(t)$ is the constant speed ${\mathcal {L}}(f(t))$ reparametrization of *f*(*t*), for all $t\in [0,T)$ we have

$$\begin{aligned} \Vert \partial _t {\tilde{f}}(t) \Vert _{L^2(\!\textrm{d}x)}\le \sqrt{\frac{2}{{\mathcal {L}}(f(t))}+16~{\mathcal {E}}(f(t))}\Vert \partial _t f \Vert _{L^2(\!\textrm{d}s_{f(t)})}. \end{aligned}$$

In particular, if *f* evolves by the length-preserving elastic flow (1.3), we have

$$\begin{aligned} \Vert \partial _t {\tilde{f}}(t) \Vert _{L^2(\!\textrm{d}x)}\le C\Vert \partial _t f \Vert _{L^2(\!\textrm{d}s_{f(t)})}, \end{aligned}$$

for all $t\in (0,T]$, where $C=\sqrt{\frac{2}{\ell }+16{\mathcal {E}}(f_0)}$.

#### Proof

Recall that by Definition 4.9 we have

$$\begin{aligned} \psi (t,\varphi (t,x)) = \varphi (t,\psi (t,x)) = x \text { for all } t\in [0,T), x\in {I}. \end{aligned}$$

For the derivatives of $\varphi$ and $\psi$ we thus obtain

(i) $$\partial _t \varphi (t,x)=-\frac{\partial _t {\mathcal {L}}(f(t))}{{\mathcal {L}}(f(t))^2} \int \nolimits _0^{x} \!\textrm{d}s_{f(t)} - \frac{1}{{\mathcal {L}}(f(t))}\int \nolimits _0^{x}\langle \partial _t f, \vec {\kappa }_{f(t)}\rangle \,\, \!\textrm{d}s_{f(t)}$$;
(ii) $$\partial _x \varphi (t,x) = \frac{|\partial _x f(t,x)|}{{\mathcal {L}}(f(t))}$$;
(iii) $$\partial _x \psi (t,\varphi (t,x)) = \left( \partial _x \varphi (t,x)\right) ^{-1} = \frac{{\mathcal {L}}(f(t))}{|\partial _x f(t,x)|}$$;
(iv) $$\begin{aligned} \partial _t \psi (t, \varphi (t,x))&= - \partial _x \psi (t,{\varphi (t,x)})~ \partial _t\varphi (t,x)\\&= \frac{{\mathcal {L}}(f(t))}{|\partial _x f(t,x)|}\left( \frac{\partial _t {\mathcal {L}}(f(t))}{{\mathcal {L}}(f(t))^2} \int \nolimits _0^{x} \!\textrm{d}s_{f(t)} \right. \\&\quad \left. + \frac{1}{{\mathcal {L}}(f(t))}\int \nolimits _0^{x}\langle \partial _t f, \vec {\kappa }_{f(t)}\rangle \,\,\!\textrm{d}s_{f(t)}\right) . \end{aligned}$$

Now, we estimate

$$\begin{aligned} \Vert \partial _t {\tilde{f}}(t) \Vert _{L^2(\!\textrm{d}x)}^2&\le 2\int \nolimits _0^1 |(\partial _t f)(t, \psi (t,x))|^2\!\textrm{d}x \\&\quad + 2\int \nolimits _0^1 |(\partial _x f)(t, \psi (t,x))|^2 ~|\partial _t \psi (t,x)|^2\!\textrm{d}x. \end{aligned}$$

Taking $y=\psi (t,x)$ and using $\psi (t,0)=0$, $\psi (t,1)=1$, we find

$$\begin{aligned} \Vert \partial _t {\tilde{f}}(t) \Vert _{L^2(\!\textrm{d}x)}^2 \le&2\Bigg (\int \nolimits _{0}^{1}|\partial _t f(t,y)|^2 \frac{1}{\partial _x \psi (t, \varphi (t,y))}\!\textrm{d}y\\&\quad {+} |\partial _x f(t,y)|^2 ~ |\partial _t \psi (t, \varphi (t,y))|^2\frac{1}{\partial _x \psi (t, \varphi (t,y))}\!\textrm{d}y\Bigg ){=:} 2(A {+} B). \end{aligned}$$

For the first integral, we clearly have

$$\begin{aligned} A = \int \nolimits _0^1 |\partial _t f(t,y)|^2 \frac{|\partial _x f(t,y)|}{{\mathcal {L}}(f(t))}\,\!\textrm{d}y = \frac{1}{{\mathcal {L}}(f(t))}\Vert \partial _t f \Vert _{L^2(\!\textrm{d}s_{f(t)})}^2. \end{aligned}$$

For the second part, note that by (iv), we have

$$\begin{aligned}&B =\int \nolimits _0^1 \Bigg \vert \frac{\partial _t {\mathcal {L}}(f(t))}{{\mathcal {L}}(f(t))} \int \nolimits _0^{y} \,\!\textrm{d}s_{f(t)} + \int \nolimits _0^{y}\langle \partial _t f, \vec {\kappa }_{f(t)}\rangle \,\!\textrm{d}s_{f(t)} \Bigg \vert ^2 \frac{|\partial _x f(t,y)|}{{\mathcal {L}}(f(t))}\,\!\textrm{d}y. \end{aligned}$$

Now, using the boundary conditions, we have $\partial _t {\mathcal {L}}(f(t)) = -\int \nolimits _{I}\langle \vec {\kappa }_{f(t)}, \partial _t f(t)\rangle \,\!\textrm{d}s_{f(t)}$ and the Cauchy–Schwarz inequality yields

$$\begin{aligned} B&\le 2 \int \nolimits _0^1 \Bigg (\left| \frac{\partial _t {\mathcal {L}}(f(t))}{{\mathcal {L}}(f(t))} \int \nolimits _0^y\,\!\textrm{d}s_{f(t)}\right| ^2 + \left| \int \nolimits _0^y \langle \partial _t f(t), \vec {\kappa }_{f(t)}\rangle \,\!\textrm{d}s_{f(t)}\right| ^2 \Bigg )\frac{|\partial _x f(t,y)|}{{\mathcal {L}}(f(t))}\,\!\textrm{d}y \\&\le 2\int \nolimits _0^1 \Bigg ( \left( \int \nolimits _0^1 |\langle \partial _t f(t), \vec {\kappa }_{f(t)}\rangle |\,\!\textrm{d}s_{f(t)}\right) ^2 \\&\quad + \left( \int \nolimits _0^1 |\langle \partial _t f(t), \vec {\kappa }_{f(t)}\rangle |\,\!\textrm{d}s_{f(t)}\right) ^2\Bigg ) \frac{|\partial _x f(t,y)|}{{\mathcal {L}}(f(t))}\,\!\textrm{d}y\\&= 4 \left( \int \nolimits _0^1 |\langle \partial _t f(t), \vec {\kappa }_{f(t)}\rangle |\,\!\textrm{d}s_{f(t)}\right) ^2 \le 4 \Vert \partial _t f(t) \Vert _{L^2(\,\!\textrm{d}s_{f(t)})}^2 \Vert \vec {\kappa }_{f(t)} \Vert _{L^2(\,\!\textrm{d}s_{f(t)})}^2 \\&= 8~ {\mathcal {E}}(f(t)) \Vert \partial _t f(t) \Vert _{L^2(\,\!\textrm{d}s_{f(t)})}^2. \end{aligned}$$

$$\square $$

#### Remark 4.11

Note that in the proof of Lemma 4.10, we only used the boundary conditions to conclude that no boundary terms appear when integrating by parts. In particular, Lemma 4.10 also holds in the case of closed curves.

Finally, we can prove our main convergence result.

#### Proof of Theorem 1.2

Let $\varepsilon >0$ and let ${\hat{f}}\in {\mathcal {C}}^{\infty }((0, \infty )\times I;{\mathbb {R}}^d)$, ${\bar{\varepsilon }}>\varepsilon$ be as in Theorem 4.1. The first statement of Theorem 1.2 follows from property (i) in Theorem 4.1, and the fact that the solution *f* in Theorem 2.9 lies in ${\mathbb {X}}_{T,2}\hookrightarrow BUC([0,T];W^{2,2}(I;{\mathbb {R}}^d))$ by Proposition B.1 and (B.1).

For the convergence statement, let ${\tilde{f}}$ be the constant speed $\ell$ reparametrization of ${\hat{f}}$, cf. Definition 4.9, and note that ${\tilde{f}}\in {\mathcal {C}}^{\infty }((0,\infty )\times I;{\mathbb {R}}^d)$. By Theorem 4.1 (iii), there exist a sequence $t_n\rightarrow \infty$ and a smooth regular curve $f_{\infty }:I\rightarrow {\mathbb {R}}^{n}$, such that ${\tilde{f}}(t_n)\rightarrow f_\infty$ in ${\mathcal {C}}^{k}(I;{\mathbb {R}}^{n})$ for all $k\in {\mathbb {N}}_0$. Moreover, as a consequence of Theorem 4.1, $f_{\infty }$ is a smooth constrained elastica, i.e. a smooth solution of (1.8).

Recall from Theorem 4.1 (ii) that ${\hat{f}}$ has tangential velocity zero for *t* sufficiently large. Thus, we can without loss of generality assume ${\mathcal {E}}({\hat{f}}(t))= {\mathcal {E}}({\tilde{f}}(t)) >{\mathcal {E}}(f_\infty )$, since otherwise ${\hat{f}}(t)$ would be eventually constant by (1.6), and hence convergent. Moreover, since ${\mathcal {E}}({\hat{f}}(t))$ is nonincreasing, we have that $\lim _{t\rightarrow \infty }{\mathcal {E}}({\hat{f}}(t)) = \lim _{n\rightarrow \infty }$ ${\mathcal {E}}({\tilde{f}}(t_n))= {\mathcal {E}}(f_\infty )$.

Since $f_{\infty }$ is smooth, by Theorem 4.8, there exist $\sigma , C_{LS}>0$ and $\theta \in (0,\frac{1}{2}]$ such that we have a refined Łojasiewicz–Simon inequality, i.e. for all $g\in {\mathcal {X}}$ satisfying $\Vert g-f_{\infty } \Vert _{W^{4,2}}\le \sigma$ we have

4.8 $$\begin{aligned} |{\mathcal {E}}(g)-{\mathcal {E}}(f_\infty )|^{1-\theta }\le C_{LS} \Vert \nabla _{L^2(\,\!\text {d}s_{g})} {\mathcal {E}}(g) + \lambda (g)\nabla _{L^2(\,\!\text {d}s_{g})} {\mathcal {L}}(g) \Vert _{L^2(\,\!\text {d}s_{g})}. \end{aligned}$$

Passing to a subsequence, we can assume $\Vert {\tilde{f}}(t_n,\cdot )-f_{\infty } \Vert _{W^{4,2}}<\sigma$ for all *n*. Define

$$\begin{aligned} s_n := \sup \left\{ s\ge t_n\mid \Vert {\tilde{f}}(t,\cdot )-f_{\infty } \Vert _{W^{4,2}}<\sigma \text { for all } t\in [t_n, s]\right\} \end{aligned}$$

and note that $$s_n>t_n$$ since $${\tilde{f}}$$ is smooth. Define $$G(t):= \left( {\mathcal {E}}({\tilde{f}}(t))-{\mathcal {E}}(f_\infty )\right) ^{\theta }$$. By our assumption $${\mathcal {E}}({\tilde{f}}(t))>{\mathcal {E}}(f_\infty )$$, so we can compute on $$[t_n, s_n)$$ using that $${\hat{f}}$$ solves (1.3) with $$\theta \equiv 0$$, so $$\partial _t {\hat{f}} = -\nabla {\mathcal {E}}({\hat{f}})-\lambda \nabla {\mathcal {L}}({\hat{f}})$$ and the fact that $${\mathcal {E}}$$ is geometric, i.e. invariant under reparametrization

$$\begin{aligned}&-\frac{\!\text {d}}{\!\text {d}t}G = \theta \left( {\mathcal {E}}({\tilde{f}} ) - {\mathcal {E}}(f_\infty )\right) ^{\theta -1}\left( -\frac{\!\text {d}}{\!\text {d}t}{\mathcal {E}}({\hat{f}} )\right) \\ {}&\quad = \theta \left( {\mathcal {E}}({\tilde{f}} ) - {\mathcal {E}}(f_\infty )\right) ^{\theta -1} \left( - \left\langle \nabla _{L^2(\,\!\text {d}s_{{\hat{f}} })}{\mathcal {E}}({\hat{f}} ), \partial _t {\hat{f}} \right\rangle _{L^2(\,\!\text {d}s_{{\hat{f}} })}\right) \\ {}&\quad = \theta \left( {\mathcal {E}}({\tilde{f}} ) - {\mathcal {E}}(f_\infty )\right) ^{\theta -1} \Vert \nabla _{L^2(\,\!\text {d}s_{{\hat{f}} })} {\mathcal {E}}({\hat{f}} ) + \lambda ({\hat{f}} )\nabla _{L^2(\,\!\text {d}s_{{\hat{f}} })} {\mathcal {L}}({\hat{f}} ) \Vert _{L^2(\,\!\text {d}s_{{\hat{f}} })} \Vert \partial _t {\hat{f}} \Vert _{L^2(\,\!\text {d}s_{{\hat{f}} })}. \end{aligned}$$

However, the quantity $\Vert \nabla _{L^2(\,\!\text {d}s_{{\hat{f}} })} {\mathcal {E}}({\hat{f}} ) +\lambda ({\hat{f}} )\nabla _{L^2(\,\!\text {d}s_{{\hat{f}} })} {\mathcal {L}}({\hat{f}} ) \Vert _{L^2(\,\!\text {d}s_{{\hat{f}} })}$ is geometric, too. Thus

$$\begin{aligned}&-\frac{\!\text {d}}{\!\text {d}t}G \\ {}&\quad {=} \,\,\theta \left( {\mathcal {E}}({\tilde{f}} ) {-} {\mathcal {E}}(f_\infty )\right) ^{\theta {-}1} \Vert \nabla _{L^2(\,\!\text {d}s_{{\tilde{f}} })} {\mathcal {E}}({\tilde{f}} ) {+} \lambda ({\tilde{f}} )\nabla _{L^2(\,\!\text {d}s_{{\tilde{f}} })} {\mathcal {L}}({\tilde{f}} ) \Vert _{L^2(\,\!\text {d}s_{{\tilde{f}} })} \Vert \partial _t {\hat{f}} \Vert _{L^2(\,\!\text {d}s_{{\hat{f}} })}\\ {}&\quad \ge \frac{\theta }{C_{LS}} \Vert \partial _t {\hat{f}} \Vert _{L^2(\,\!\text {d}s_{{\hat{f}} })}. \end{aligned}$$

on $$[t_n, s_n)$$ by (4.8) and our choice of $$s_n$$. Therefore, by Lemma 4.10 we have

4.9 $$\begin{aligned} -\frac{\!\text {d}}{\!\text {d}t}G(t) \ge {C} \Vert \partial _t {\tilde{f}} \Vert _{L^2(\,\!\text {d}x)},\end{aligned}$$

for all $t\in [t_n, s_n)$, where ${C} = C(\ell , {\mathcal {E}}(f_0), \theta , C_{LS})>0$. Let $t\in [t_n, s_n)$. Then

4.10 $$\begin{aligned} \Vert {\tilde{f}}(t)-{\tilde{f}}(t_n) \Vert _{L^2(\,\!\text {d}x)}\le \int \nolimits _{t_n}^{t}\Vert \partial _t {\tilde{f}}(\tau ) \Vert _{L^2(\,\!\text {d}x)}\,\,\text {d}\tau \le \frac{1}{{C}} G(t_n)\rightarrow 0 \end{aligned}$$

using (4.9) and ${\mathcal {E}}({\tilde{f}}(t_n)) \rightarrow {\mathcal {E}}(f_\infty )$ as $n\rightarrow \infty$. We now assume that all of the $s_n$ are finite. Then, by continuity (4.10) also holds for $t=s_n$. By the subconvergence result in Theorem 4.1, passing to a subsequence we have ${\tilde{f}}(s_{n})\rightarrow \psi$ smoothly as $n \rightarrow \infty$. Moreover, by continuity and the definition of $s_n$, we have that $\Vert \psi - f_\infty \Vert _{W^{4,2}}=\sigma$, whereas $\Vert \psi -f_\infty \Vert _{L^2(\,\!\text {d}x)} = \lim _{n\rightarrow \infty }\Vert {\tilde{f}}(s_{n})-{\tilde{f}}(t_{n}) \Vert _{L^2(\,\!\text {d}x)} = 0$ by (4.10), a contradiction.

Consequently, there has to exist some $n_0\in {\mathbb {N}}$ such that $s_{n_0}=\infty$, and this yields $\Vert {\tilde{f}}(t)-f_\infty \Vert _{W^{4,2}}<\sigma$ for all $t\ge t_{n_0}$. This means that (4.9) holds for any $t\ge t_{n_0}$; thus, $t\mapsto \Vert \partial _t {\tilde{f}}(t) \Vert _{L^2(\,\!\text {d}x)}\in L^1([0,\infty );{\mathbb {R}})$. Hence, for all $t_{n_0}\le t\le t^{\prime }$ we have

$$\begin{aligned} \Vert {\tilde{f}}(t) - {\tilde{f}}(t^{\prime }) \Vert _{L^2(\,\!\text {d}x)} \le \int \nolimits _{t}^{t^{\prime }}\Vert \partial _t{\tilde{f}}(\tau ) \Vert _{L^2(\,\!\text {d}x)}\,\,\text {d}\tau \rightarrow 0, \end{aligned}$$

as $t,t'\rightarrow \infty$ by the dominated convergence theorem. Therefore, $\lim _{t\rightarrow \infty }{\tilde{f}}(t)$ exists in $L^2(\,\!\text {d}x)$ and thus equals $f_\infty$. A subsequence argument shows that for any $k\in {\mathbb {N}}_0$ we have $\Vert {\tilde{f}}(t)-f_\infty \Vert _{{\mathcal {C}}^{k}(I;{\mathbb {R}}^d)}\rightarrow 0$ as $t\rightarrow \infty$, i.e. the convergence is smooth. $\square$

## Data Availability

Data sharing is not applicable to this article as no datasets were generated or analysed during the current study.

## Appendix A: Explicit formulas in coordinates

In this section, we present the explicit representation of the geometric quantities appearing in this article. They can be obtained by a straight forward calculation, see e.g. [20, (2.3)].

### Proposition A.1

Suppose $f:I \rightarrow {\mathbb {R}}^{d}$ is a smooth immersion. With the arc-length element $\gamma = |\partial _x f|$ we have

(i) $$\vec {\kappa }_f = \partial _s^{2} f = \frac{\partial ^2_x f}{\gamma ^{2}} - \frac{\langle \partial _x^{2}f, \partial _x f\rangle }{\gamma ^{4}} \partial _x f = \frac{(\partial _x^2 f)^{\perp }}{\gamma ^4}$$;
(ii) $$\nabla _{s_f} \vec {\kappa }_f = \frac{\partial _x^{3}f}{\gamma ^{3}} - \frac{\langle \partial _x^{3}f, \partial _x f\rangle }{\gamma ^{5}}\partial _x f - 3 \frac{\langle \partial _x^{2} f, \partial _{x} f\rangle }{\gamma ^{5}} \partial _x^{2} f + 3 \frac{\langle \partial _x^{2}f, \partial _{x}f\rangle ^{2}}{\gamma ^{7}}\partial _x f$$;
(iii) $$\begin{aligned} \nabla _{s_f}^2 \vec {\kappa }_f&= \Bigg [ \frac{\partial _x^{4}f}{\gamma ^{4}} -6 \frac{\langle \partial _{x}^{2} f, \partial _x f\rangle }{\gamma ^{6}}\partial _x^{3}f - 4 \frac{\langle \partial _x^{3}f, \partial _x f\rangle }{\gamma ^{6}}\partial _x^{2}f \\&\quad - 3 \frac{|\partial _x^{2} f|^{2}}{\gamma ^{6}} \partial _x^{2} f + 18 \frac{\langle \partial _x^{2}f, \partial _x f\rangle ^{2}}{\gamma ^{8}} \partial _x^{2}f\Bigg ]^{\perp _f}; \end{aligned}$$
(iv) $$\begin{aligned} \nabla {\mathcal {E}}(f)&=\Bigg [\frac{\partial _x^{4}f}{\gamma ^{4}} -6 \frac{\langle \partial _{x}^{2} f, \partial _x f\rangle }{\gamma ^{6}}\partial _x^{3}f - 4 \frac{\langle \partial _x^{3}f, \partial _x f\rangle }{\gamma ^{6}}\partial _x^{2}f \\&\quad - \frac{5}{2} \frac{|\partial _x^{2} f|^{2}}{\gamma ^{6}} \partial _x^{2} f + \frac{35}{2} \frac{\langle \partial _x^{2}f, \partial _x f\rangle ^{2}}{\gamma ^{8}} \partial _x^{2}f\Bigg ]^{\perp _f}.\end{aligned}$$

Here $\nabla {\mathcal {E}}(f)$ denotes the $L^2(\!\textrm{d}s_f)$-gradient of ${\mathcal {E}}$ at *f*.

### Lemma A.2

Let *f* be a smooth immersed curve with arc-length element $\gamma$. Then

(i) $$|\vec {\kappa }_{f}|^{4} = \gamma ^{-8} |\partial _x^2 f|^{4} - 2\gamma ^{-10} |\partial _x^{2}f|^{2}\langle \partial _x^{2}f, \partial _x f\rangle ^{2} + \gamma ^{-12} \langle \partial _x^{2}f, \partial _x f\rangle ^{4}$$;
(ii) $$\begin{aligned} |\nabla _{s_f}\vec {\kappa }_{f}|^{2}&= \gamma ^{-6}|\partial _x^{3} f|^{2} - \gamma ^{-8} \langle \partial _x^{3}f, \partial _x f\rangle ^{2} - 6\gamma ^{-8}\langle \partial _x^{3}f, \partial _x^{2}f\rangle \langle \partial _x^{2}f, \partial _x f\rangle \\&\quad + 6 \gamma ^{-10} \langle \partial _x^{3}f, \partial _x f\rangle \langle \partial _x^{2}f, \partial _x f\rangle ^{2} + 9 \gamma ^{-10}\langle \partial _x^{2}f, \partial _x f\rangle ^{2} |\partial _x^{2}f|^{2} \\&\quad - 9 \gamma ^{-12}\langle \partial _x^{2}f, \partial _x f\rangle ^{4}; \end{aligned}$$
(iii) $$\begin{aligned} \langle \nabla _{s_f}\vec {\kappa }_{{f}}, \vec {\kappa }_f\rangle&= \gamma ^{-5} \langle \partial _x^{3}f, \partial _x^{2}f\rangle - \gamma ^{-7} \langle \partial _x^{3}f, \partial _x f\rangle \langle \partial _x^{2}f, \partial _x f\rangle \\&\quad - 3\gamma ^{-7}\langle \partial _x^{2}f, \partial _x f\rangle |\partial _x^{2} f|^2 + 3\gamma ^{-9} \langle \partial _x^{2}f, \partial _x f\rangle ^{3}. \end{aligned}$$

## Appendix B: Function spaces

In this section, we collect all relevant information on the function spaces for maximal $L^p$-regularity. Most of the embedding results are collected in [16], see also [32], where even a polynomial weight $t^{1-\mu }$ in time is allowed. As discussed in Remark 2.11, time weights do not allow to prove short-time existence with weaker initial data, and hence we restrict ourselves to the case $\mu =1$.

Let $J\subset {\mathbb {R}}$ be an interval, $1\le p<\infty$. For any $s\in (0,\infty ){\setminus }{\mathbb {N}}$ and a Banach space *E*, the (*E*-valued) *Sobolev–Slobodetskii space* and the *Bessel potential space*, respectively, are given by real and complex interpolation

$$\begin{aligned} W^{s,p}(J;E)&:=\left( W^{[s],p}(J;E), W^{[s]+1,p}(J;E)\right) _{s-[s],p};\\ H^{s,p}(J;E)&:=\left( W^{[s],p}(J;E), W^{[s]+1,p}(J;E)\right) _{s-[s]}; \end{aligned}$$

where $W^{k,p}(J;E)$ denotes the usual Bochner–Sobolev space for $k\in {\mathbb {N}}_0$. Recall from [51, Theorem 2.4.1 (a), Definition 4.2.1] that the *Besov spaces* are given by

$$\begin{aligned} B^{s}_{p,q}(I;{\mathbb {R}}^d) :=\left( W^{m_1,p}(I;{\mathbb {R}}^d), W^{m_2,p}(I;{\mathbb {R}}^d)\right) _{\theta ,q}, \end{aligned}$$

where $s=(1-\theta )m_1+\theta m_2$, $\theta \in (0,1), m_1, m_2\in {\mathbb {N}}_0, m_1<m_2, p,q\in [1,\infty )$. By [51, Definition 2.3.1 (d) and Theorem 2.3.2 (d)] we have the relation

B.1 $$\begin{aligned} B^{s}_{p,p}(I;{\mathbb {R}}^d)&=W^{s,p}(I;{\mathbb {R}}^d) \quad \text { for }s\not \in {\mathbb {N}};\nonumber \\ B^{s}_{2,2}(I;{\mathbb {R}}^d)&=W^{s,2}(I;{\mathbb {R}}^d) \quad \text { for }s>0. \end{aligned}$$

Moreover, recall from [16, Section 2], that in the setting of the maximal regularity spaces ${\mathbb {X}}_{T,p}$, the spaces of zeroth- and first-order boundary data are given by

B.2 $$\begin{aligned} {\mathcal {D}}^0_{T,p}&:=W^{1-\frac{1}{4p},p}(0,T;L^p(\partial I;{\mathbb {R}}^d)) \cong W^{1-\frac{1}{4p},p}(0,T;({\mathbb {R}}^d)^2); \nonumber \\ {\mathcal {D}}^1_{T,p}&:=W^{\frac{3}{4}-\frac{1}{4p},p}(0,T;L^p(\partial I;{\mathbb {R}}^d)) \cong W^{\frac{3}{4}-\frac{1}{4p},p}(0,T;({\mathbb {R}}^d)^2). \end{aligned}$$

We now recall the Sobolev embeddings for the maximal regularity space ${\mathbb {X}}_{T,p}$. We emphasize that the operator norms of the embeddings below might blow up as $T\rightarrow 0+$. However, in the solution space with vanishing trace, i.e. the space ${}_{0}{{\mathbb {X}}_{T,p}}$, they are bounded independently of *T*.

### Proposition B.1

Let $0<T\le \infty$, let $p\ge 2$ and let $k\in \{0,\dots ,4\}$.

(i) ${\mathbb {X}}_{T,p} \hookrightarrow BUC([0,T], B^{4(1-\frac{1}{p})}_{p,p})(I;{\mathbb {R}}^d))$ with the estimate
  $$\begin{aligned} \Vert f \Vert _{BUC([0,T];B^{4(1-\frac{1}{p})}_{p,p}(I;{\mathbb {R}}^d))} \le {C(p)}\Vert f \Vert _{{\mathbb {X}}_{T,p}} \quad \text {for }f\in {}_{0}{{\mathbb {X}}_{T,p}}. \end{aligned}$$
(ii) ${\mathbb {X}}_{T,p} \hookrightarrow {\mathcal {C}}^{\alpha }([0,T];{\mathcal {C}}^{1,\alpha }(I;{\mathbb {R}}^d))$ for some $\alpha \in (0,1)$ with the estimate
  $$\begin{aligned} \Vert f \Vert _{{\mathcal {C}}^{\alpha }([0,T]; {\mathcal {C}}^{1,\alpha }(I;{\mathbb {R}}^d))}\le {C(p,\alpha )} \Vert f \Vert _{{\mathbb {X}}_{T,p}}\quad \text {for }f\in {}_{0}{{\mathbb {X}}_{T,p}}. \end{aligned}$$
(iii) The *k*-th spatial derivative is continuous as a map
  $$\begin{aligned} \partial _x^k :{\mathbb {X}}_{T,p}&\rightarrow H^{\frac{4-k}{4}, p}(0,T;L^p(I;{\mathbb {R}}^d)) \cap L^p(0,T; H^{4-k,p}(I;{\mathbb {R}}^d)) \\&\hookrightarrow W^{\frac{(4-k)\theta }{4}, p}(0,T;W^{(4-k)(1-\theta ),p}(I;{\mathbb {R}}^d)) \text { for all } \theta \in (0,1), \end{aligned}$$
  with the estimate
  $$\begin{aligned} \Vert \partial _x^k f \Vert _{H^{\frac{4-k}{4}, p}(0,T;L^p(I;{\mathbb {R}}^d)) \cap L^p(0,T; H^{4-k,p}(I;{\mathbb {R}}^d))}\le {C(k,p)} \Vert f \Vert _{{\mathbb {X}}_{T,p}}\quad \text {for }f\in {}_{0}{{\mathbb {X}}_{T,p}}. \end{aligned}$$
(iv) The spatial trace of the *k*-th spatial derivative is continuous as a map
  $$\begin{aligned} {{\,\textrm{tr}\,}}_{\partial I}\partial _x^k :{\mathbb {X}}_T&\rightarrow W^{\frac{4-k}{4} - \frac{1}{8}, p}(0,T;L^p(\partial I;{\mathbb {R}}^d)) \cong W^{\frac{4-k}{4} - \frac{1}{8}, p}(0,T;({\mathbb {R}}^d)^2), \end{aligned}$$
  with the estimate
  $$\begin{aligned} \Vert {{\,\textrm{tr}\,}}_{\partial I}\partial _x^k f \Vert _{W^{\frac{4-k}{4} - \frac{1}{8}, p}(0,T;({\mathbb {R}}^d)^2)}\le {C(k,p)} \Vert f \Vert _{{\mathbb {X}}_{T,p}}\quad \text {for }f\in {}_{0}{{\mathbb {X}}_{T,p}}. \end{aligned}$$

### Proof

For $T=\infty$ and *I* replaced by ${\mathbb {R}}$, the statements follow from the corresponding results in [16, Section 3]. The statements in our case can then be obtained by considering appropriate temporal and spatial extension operators, see for instance [32, Lemma 2.5 and (3.2)]. When restricted to ${}_{0}{{\mathbb {X}}_{T,p}}$, the operator norm of the temporal extension does not depend on *T* by [32, Lemma 2.5], which implies the above *T*-independent estimates. $\square$

### Remark B.2

For a Hilbert space *E* and $s\in (0,\infty ),p=2$, the Bessel potential spaces coincide with the Slobodetskii spaces, i.e. $H^{s,2}(0,T;E) = W^{s,2}(0,T;E)$ with equivalence of norms, cf. [29, Corollary 4.37]. A particular consequence of this is that in the case $p=2$ we get from Proposition B.1 (iii) that for $k\in {\mathbb {N}}$, $k\le 4$, and $T\in (0,1]$ we have

$$\begin{aligned} \partial _x^k :{\mathbb {X}}_{T,2}&\rightarrow W^{\frac{4-k}{4}, 2}(0,T;L^2(I;{\mathbb {R}}^d)) \cap L^2(0,T; W^{4-k,2}(I;{\mathbb {R}}^d)) \end{aligned}$$

is continuous, with the estimate

$$\begin{aligned} \Vert \partial _x^k f \Vert _{W^{\frac{4-k}{4}, 2}(0,T;L^2(I;{\mathbb {R}}^d)) \cap L^2(0,T; W^{4-k,2}(I;{\mathbb {R}}^d))}\le {C(k)} \Vert f \Vert _{{\mathbb {X}}_{T,2}}\quad \text {for }f\in {}_{0}{{\mathbb {X}}_{T,2}}. \end{aligned}$$

A crucial tool in proving the contraction estimates in Sect. 2.4 is the precise control of the integrability of the spatial derivatives and their spatial trace, with operator norm bounded independent of *T*. As in Sect. 2.4, we restrict to the case $p=2$ here.

### Proposition B.3

Let $$T\in (0,1]$$, $$k\in {\mathbb {N}}$$, $$k\le 4$$, $$\rho _1, \rho _2 \in [1, \infty )$$.

(i) If there exists $\theta \in [0,1]$ such that $\frac{4-k}{4}\theta - \frac{1}{2}\ge -\frac{1}{\rho _1}$ and $(4-k)(1-\theta )-\frac{1}{2}\ge -\frac{1}{\rho _2}$ then $\partial _x^k :{{\mathbb {X}}_{T,2}}\rightarrow L^{\rho _1}(0,T;L^{\rho _2}(I;{\mathbb {R}}^d))$ with the estimate
  $$\begin{aligned} \Vert \partial _x^k f \Vert _{L^{\rho _1}(0,T;L^{\rho _2}(I;{\mathbb {R}}^d))} \le {C(k,\theta , \rho _1, \rho _2)}\Vert f \Vert _{{\mathbb {X}}_{T,2}} \quad \text { for all }f\in {}_{0}{{\mathbb {X}}_{T,2}}. \end{aligned}$$
(ii) If $\frac{4-k}{4}-\frac{5}{8}\ge -\frac{1}{\rho _1}$, then ${{\,\textrm{tr}\,}}_{\partial I} \partial _x^k :{{\mathbb {X}}_{T,2}}\rightarrow L^{\rho _1}(0,T;({\mathbb {R}}^d)^2)$ with the estimate
  $$\begin{aligned} \Vert {{\,\textrm{tr}\,}}_{\partial I}\partial _x^k f \Vert _{L^{\rho _1}(0,T;({\mathbb {R}}^d)^2} \le {C(k, \rho _1)}\Vert f \Vert _{{\mathbb {X}}_{T,2}} \quad \text { for all }f\in {}_{0}{{\mathbb {X}}_{T,2}}. \end{aligned}$$

### Proof

We first prove the estimates.

(i) Using first Proposition B.1 (iii) and Remark B.2, then interpolation, and in the last line the usual Sobolev embedding both in the temporal and spatial variable, we find
  B.3 $$\begin{aligned} \partial _x^k :{}_{0}{{\mathbb {X}}_{{1},2}}&\rightarrow W^{\frac{4-k}{4},2}(0,{1};L^2(I;{\mathbb {R}}^d)) \cap L^2(0,{1}, W^{4-k, 2}(I;{\mathbb {R}}^d)) \nonumber \\&\hookrightarrow W^{\frac{4-k}{4}\theta ,2}(0,{1};W^{(4-k)(1-\theta ),2}(I;{\mathbb {R}}^d)) \nonumber \\&\hookrightarrow L^{\rho _1}(0, {1}; L^{\rho _2}(I;{\mathbb {R}}^d)). \end{aligned}$$
  Now, by [32, Lemma 2.5], there exists an extension operator $E_T$ from (0, *T*) to $(0,{1})$ such that $E_T :{}_{0}{{\mathbb {X}}_{T,2}} \rightarrow {}_{0}{{\mathbb {X}}_{{1},2}}$ has operator norm independent of *T*. Then, for any $f\in {}_{0}{{\mathbb {X}}_{T,2}}$ we have using (B.3)
  $$\begin{aligned} \Vert \partial _x^k f \Vert _{L^{\rho _1}(0,T;L^{\rho _2}(I;{\mathbb {R}}^d))}&\le \Vert \partial _x^k(E_T f) \Vert _{L^{\rho _1}(0,{1};L^{\rho _2}(I;{\mathbb {R}}^d))} \\&\le {C(k, \theta , \rho _1, \rho _2)} \Vert E_T f \Vert _{{\mathbb {X}}_{{1},2}} \\&\le {C(k, \theta , \rho _1, \rho _2)} \Vert f \Vert _{{\mathbb {X}}_{T,2}}. \end{aligned}$$
(ii) Since ${{\,\textrm{tr}\,}}_{\partial I}\partial _k f$ only depends on the temporal variable, we first use Proposition B.1 (iv) and Remark B.2 and then the Sobolev embedding to find
  B.4 $$\begin{aligned} {{\,\textrm{tr}\,}}_{\partial I}\partial _x^k :{}_{0}{{\mathbb {X}}_{{1},2}}&\rightarrow W^{\frac{4-k}{4}-\frac{1}{8},2}(0,{1};({\mathbb {R}}^d)^2)\nonumber \\&\hookrightarrow L^{\rho _1}(0, {1}; ({\mathbb {R}}^d)^2). \end{aligned}$$
  Again, using the extension operator, we find for any $f\in {}_{0}{{\mathbb {X}}_{T,2}}$
  $$\begin{aligned} \Vert {{\,\textrm{tr}\,}}_{\partial I}\partial _x^k f \Vert _{L^{\rho _1}(0,T;({\mathbb {R}}^d)^2)}&\le \Vert {{\,\textrm{tr}\,}}_{\partial I}\partial _x^k(E_T f) \Vert _{L^{\rho _1}(0,{1};({\mathbb {R}}^d)^2)} \\&\le {C(k, \rho _1)} \Vert E_T f \Vert _{{\mathbb {X}}_{{1},2}} \\&\le {C(k, \rho _1)} \Vert f \Vert _{{\mathbb {X}}_{T,2}}. \end{aligned}$$

The mapping properties follow from (B.3) and (B.4). $\square$

## Appendix C: Details of the contraction estimates

First, the following definition describes the structure of the nonlinearities in (2.3) which guarantees the desired contraction properties.

### Definition C.1

Let $(a,b)\in {\mathbb {N}}_0^{2}$. We denote by $A^{(a,b)}$ the set of bounded multilinear maps

C.1 $$\begin{aligned} \varphi :({\mathbb {R}}^d)^{m}\times ({\mathbb {R}}^d)^{a}\times ({\mathbb {R}}^d)^{b}\rightarrow {\mathbb {R}}^{w} \end{aligned}$$

for some $w\in {\mathbb {N}}$, $m\in {\mathbb {N}}_0$. Then, we define the set ${\mathcal {A}}^{(a,b)}$ of *multilinear maps of type (a, b)* as the set of all maps $f\mapsto \Phi (f)$ acting via

$$\begin{aligned}&\Phi (f)(t,x) \\&\quad = \varphi \Big (\underbrace{\partial _x f(t,x), \dots , \partial _x f(t,x)}_{m\text {-times}}, \underbrace{\partial _x^{2}f(t,x), \dots , \partial _x^{2}f(t,x)}_{a \text {-times}}, \underbrace{\partial _x^{3}f(t,x), \dots , \partial _x^{3}f(t,x)}_{b\text {-times}}\Big ), \end{aligned}$$

for almost every $(t,x)\in (0,T)\times I$ where $\varphi \in A^{(a,b)}$.

### Remark C.2

Note that we do not keep track of *m*, the number of first-order derivatives appearing in $\Phi \in {\mathcal {A}}^{(a,b)}$. This is justified since by Proposition B.1 (ii), the derivatives of first order of $f\in {\mathbb {X}}_{T}$ are in ${\mathcal {C}}([0,T]\times I;{\mathbb {R}}^d)$ and hence do not affect the integrability of $\Phi (f)$.

### Example C.3

The map $f\mapsto \Phi (f) = {\langle \partial _x^2f, \partial _x f\rangle } \partial _x^3 f$ is in ${\mathcal {A}}^{(1,1)}$, since the derivatives of second and third order only appear linearly.

The following proposition yields for which parameters (*a*, *b*) we get a contraction. Note that nonlinearities with this structure appear in ${\tilde{F}}$ in (2.2) and $\lambda$ in (2.1). As in Sect. 2.4, we assume $T,M\le 1$ and set ${\mathbb {X}}_T = {\mathbb {X}}_{T,2}$ to simplify notation.

### Proposition C.4

Let $q\in (0,1)$ and let $\Phi \in {\mathcal {A}}^{(a,b)}$. Then, for $T=T(q,{\bar{f}}),M=M(q,{\bar{f}})\in (0,1]$ small enough, each of the following nonlinear maps is a well-defined *q*-contraction, i.e. Lipschitz continuous with Lipschitz constant *q*.

(i) $${{\bar{B}}_{T,M}}\rightarrow L^2(0,T;L^2), f\mapsto \Phi (f)$$, if $$(a,b)=(1,1)$$ or $$(a,b)=(3,0)$$.
(ii) ${{\bar{B}}_{T,M}} \rightarrow L^2(0,T), f\mapsto \int \nolimits _{I} \Phi (f)\!\textrm{d}x$, if $(a,b)=(0,2), (a,b)=(2,1)$ or $(a,b)=(4,0)$.
(iii) ${{\bar{B}}_{T,M}} \rightarrow L^2(0,T;({\mathbb {R}}^{d})^2), f\mapsto {{\,\textrm{tr}\,}}_{\partial I} \Phi (f)$, if $(a,b)=(1,1)$ or $(a,b)=(3,0)$.
(iv) $${{\bar{B}}_{T,M}} \rightarrow L^2(0,T;L^2), f\mapsto \left( \gamma _0^{-4}-\gamma ^{-4}\right) \partial _x^4f$$.

The following general functional analytic result gives sufficient conditions for a multilinear map to be a *q*-contraction for $T>0$ small. It is the key ingredient in the proof of Proposition C.4.

### Lemma C.5

Let $1\le q_1\le \infty$ and suppose $(f_1, \dots , f_r)\mapsto \mu (f_1, \dots , f_r)$ is a multilinear map such that for all $f_1, \dots , f_r\in {\mathbb {X}}_{T,2}$ we have

C.2 $$\begin{aligned} \Vert \mu (f_1, \dots , f_r) \Vert _{L^{q_1}(0,T;Z)}\le C \prod _{j=1}^{r} \Vert S\partial _x^{d_j} f_j \Vert _{X_j}. \end{aligned}$$

Here, we have $d_1, \dots , d_r \in \{0,\dots , 3\}$, $S\in \{{{\,\textrm{Id}\,}}, {{\,\textrm{tr}\,}}_{\partial I}\}$ and $Z,X_1, \dots , X_r$ are Banach spaces such that there exists $C\in (0,\infty )$ independent of *T* with

(i) $\partial _x^{d_i} :{\mathbb {X}}_T \rightarrow X_i$ and for $f\in {}_{0}{{\mathbb {X}}_T}$ we have $\Vert S \partial _x^{d_i}f \Vert _{X_i}\le {C}\Vert f \Vert _{{\mathbb {X}}_T}$ for all $i=1, \dots ,r$.
(ii) for all $j=1, \dots r$ one of the following conditions is satisfied.
  - There exists $\alpha >0$ with $\Vert S\partial _x^{d_j}f \Vert _{X_j}\le {C} T^{\alpha } \Vert f \Vert _{{\mathbb {X}}_T}$ for all $f\in {}_{0}{{\mathbb {X}}}_T$.
  - There exists $k\ne j$ with $\Vert S\partial _x^{d_k}f \Vert _{X_k}\rightarrow 0$ as $T\rightarrow 0$ for all $f\in {\mathbb {X}}_T$.

Then, setting $\mu (f) = \mu (f, \dots , f)$, we have $\mu (f)\in L^{q_1}(0,T;Z)$ for all $f\in {\mathbb {X}}_T$ and for any $q\in (0,1)$, there exist $M = M(q,r,{\bar{f}}),T = T(q,r, {\bar{f}})\in (0,1]$ small enough, such that for all $f,{\tilde{f}}\in {{\bar{B}}_{T,M}}$ we have

$$\begin{aligned} \Vert \mu (f)-\mu ({\tilde{f}}) \Vert _{L^{q_1}(0,T;Z)}\le q\Vert f-{\tilde{f}} \Vert _{{\mathbb {X}}_T}. \end{aligned}$$

### Remark C.6

When applying Lemma C.5, we always work with Banach spaces of the type $X_j=L^{p_j}(0,T;L^{q_j})$ and $Z=L^{p_0}$, for some $p_0, p_j, q_j \in [1,\infty ]$. Note that (ii) b) is always satisfied if there exists $k\ne j$ with $p_k<\infty$, since then $\lim _{T\rightarrow 0} \Vert f \Vert _{L^{p_k}(0,T;L^{q_k})}\rightarrow 0$ by dominated convergence.

### Proof of Lemma C.5

Let $f, {\tilde{f}}\in {{\bar{B}}_{T,M}}$. Adding and subtracting zeroes and using the multilinearity, we get

$$\begin{aligned} \mu (f)-\mu ({\tilde{f}})&= \mu (f-{\tilde{f}},f,\dots , f) + \mu ({\tilde{f}}, f-{\tilde{f}}, f, \dots , f)\\&\quad + \dots + \mu ({\tilde{f}}, \dots , f-{\tilde{f}}, f)+\mu ({\tilde{f}}, \dots , {\tilde{f}}, f-{\tilde{f}}). \end{aligned}$$

Thus, using (C.2), we get

C.3 $$\begin{aligned}&\Vert \mu (f)-\mu ({\tilde{f}}) \Vert _{L^{q_1}(0,T;Z)}\nonumber \\&\quad \le C\sum _{j=1}^r \Vert S\partial _x^{d_1}{\tilde{f}} \Vert _{X_1} \dots \Vert S \partial _x^{d_j}(f-{\tilde{f}}) \Vert _{X_j} \dots \Vert S\partial _x^{d_r}f \Vert _{X_r}. \end{aligned}$$

We now show that the contraction property is valid for each summand in (C.3). Note that for all $k\in \{1, \dots ,r\}$ by (i) we have

C.4 $$\begin{aligned} \Vert S\partial _x^{d_k}f \Vert _{X_k}&\le \Vert S\partial _x^{d_k}(f-{\bar{f}}) \Vert _{X_k} + \Vert S\partial _x^{d_k}{\bar{f}} \Vert _{X_k}\nonumber \\&\le {C}\Vert f-{\bar{f}} \Vert _{{\mathbb {X}}_T} + \Vert S\partial _x^{d_k}{\bar{f}} \Vert _{X_k} \le {C}\left( M + \Vert S\partial _x^{d_k}{\bar{f}} \Vert _{X_k}\right) . \end{aligned}$$

In particular, for $T\le 1$, $M\le 1$ we find

C.5 $$\begin{aligned} \Vert S\partial _x^{d_k}f \Vert _{X_k}, \Vert S\partial _x^{d_k}{\tilde{f}} \Vert _{X_k}\le {C({\bar{f}})}. \end{aligned}$$

Now, let $j\in \{1, \dots , r\}$. If (ii) a) is satisfied, using $f(0)={\tilde{f}}(0)=f_0$, we find

$$\begin{aligned}&\Vert S\partial _x^{d_1}{\tilde{f}} \Vert _{X_1} \dots \Vert S\partial _x^{d_{j-1}}{\tilde{f}} \Vert _{X_{j-1}}\Vert S\partial _x^{d_j}(f-{\tilde{f}}) \Vert _{X_j}\Vert S\partial _x^{d_{j+1}}f \Vert _{X_{j+1}}\dots \Vert S\partial _x^{d_r}f \Vert _{X_r}\\&\quad \le {C({\bar{f}})} T^{\alpha }\Vert f-{\tilde{f}} \Vert _{{\mathbb {X}}_T} \le \frac{q}{Cr} \Vert f-{\tilde{f}} \Vert _{{\mathbb {X}}_T}, \end{aligned}$$

for $T = T(\alpha , {\bar{f}})>0$ small enough. Otherwise, if (ii) b) is satisfied, we estimate using (i) for the *j*-th factor, (C.4) for the *k*-th factor and (C.5) for the remaining factors, to get

$$\begin{aligned}&\Vert S\partial _x^{d_1}{\tilde{f}} \Vert _{X_1} \dots \Vert S\partial _x^{d_{j-1}}{\tilde{f}} \Vert _{X_{j-1}}\Vert S \partial _x^{d_j}(f-{\tilde{f}}) \Vert _{X_j}\Vert S\partial _x^{d_{j+1}}f \Vert _{X_{j+1}}\dots \Vert S\partial _x^{d_r}f \Vert _{X_r}\\&\quad \le {C({\bar{f}})} \left( M + \Vert S\partial _x^{d_k}{\bar{f}} \Vert _{X_k}\right) \Vert f-{\tilde{f}} \Vert _{{\mathbb {X}}_T}. \end{aligned}$$

By (ii) b), $\lim _{T\rightarrow 0}\Vert S\partial _x^{d_k}{\bar{f}} \Vert _{X_k} = 0$. Consequently, for ${T = T(q,r,{\bar{f}}), M =}$ ${M(q,r, {\bar{f}}) \in (0,1]}$ small enough we find

$$\begin{aligned}&\Vert S\partial _x^{d_1}{\tilde{f}} \Vert _{X_1} \dots \Vert S\partial _x^{d_{j-1}}{\tilde{f}} \Vert _{X_{j-1}}\Vert S \partial _x^{d_j}(f-{\tilde{f}}) \Vert _{X_j}\Vert S\partial _x^{d_{j+1}}f \Vert _{X_{j+1}}\dots \Vert S\partial _x^{d_r}f \Vert _{X_r} \\&\quad \le \frac{q}{Cr}\Vert f-{\tilde{f}} \Vert _{{\mathbb {X}}_T}. \end{aligned}$$

All in all, we have proven

$$\begin{aligned} \Vert \mu (f)-\mu ({\tilde{f}}) \Vert _{L^{q_1}(0,T;Z)}\le q \Vert f-{\tilde{f}} \Vert _{{\mathbb {X}}_T}\quad \text { for }f, {\tilde{f}}\in {{\bar{B}}_{T,M}}.&\end{aligned}$$

$$\square $$

Together with the embedding results in Proposition B.1 and Proposition B.3, we can now prove Proposition C.4.

### Proof of Proposition C.4

Let $f, {\tilde{f}}\in {{\bar{B}}_{T,M}}\subset {\mathbb {X}}_T$ with ${T=T(q,{\bar{f}}), M=M(q,{\bar{f}})}$ ${\in (0,1]}$ small enough such that Lemma 2.2 is satisfied. The strategy for the proof of cases (i)–(iii) is to apply Lemma C.5. To that end, we use Hölder’s inequality in time and space and then verify the assumptions of Lemma C.5 using Proposition B.3. In the following, we denote by $f_1,\dots ,f_m$, $g,g_1, g_2, g_3, g_4, h, h_1, h_2$ general functions in ${{\bar{B}}_{T,M}}$.

Case (i): If $(a,b)=(1,1)$, by Hölder’s inequality we have

C.6 $$\begin{aligned}&\Vert \varphi (\partial _x f_1, \dots , \partial _x f_m, \partial _x^2g, \partial _x^3h) \Vert _{L^2(0,T;L^2)}\nonumber \\&\quad \le C(\varphi )\prod _{j=1}^m\Vert \partial _x f_j \Vert _{\infty }\Vert \partial _x^2g \Vert _{L^{4}(0,T;L^{8})} \Vert \partial _x^3 h \Vert _{L^{4}(0,T;L^{\frac{8}{3}})}. \end{aligned}$$

Using Proposition B.1 (ii) we have

$$\begin{aligned}&\partial _x :{\mathbb {X}}_T \rightarrow {\mathcal {C}}^{\alpha }([0,T]; {\mathcal {C}}(I;{\mathbb {R}}^d)),\nonumber \\&\text {with the estimate } \Vert \partial _x f \Vert _{{\mathcal {C}}^{\alpha }([0,T];{\mathcal {C}}(I;{\mathbb {R}}^d))}\le {C}\Vert f \Vert _{{\mathbb {X}}_T} \text { for }f\in {}_{0}{{\mathbb {X}}_{T}}. \end{aligned}$$

Therefore, we find

C.7 $$\begin{aligned}&\partial _x :{\mathbb {X}}_T \rightarrow {\mathcal {C}}([0,T]\times I;{\mathbb {R}}^d),\nonumber \\&\text {with the estimate } \Vert \partial _x f \Vert _{\infty }\le {C}T^{\alpha }\Vert f \Vert _{{\mathbb {X}}_T} \text { for }f\in {}_{0}{{\mathbb {X}}_{T}}, \end{aligned}$$

such that (ii) a) in Lemma C.5 is satisfied. Next, using Proposition B.3 (i) with $k=2$ and $\theta =\frac{3}{4}$ yields

C.8 $$\begin{aligned}&\partial _x^2 :{\mathbb {X}}_T \rightarrow L^8(0,T;L^4), \nonumber \\&\text {with the estimate } \Vert \partial _x^2 f \Vert _{L^8(0,T;L^4)}\le {C}\Vert f \Vert _{{\mathbb {X}}_T} \quad \text { for all }f\in {}_{0}{{\mathbb {X}}_{T}}, \end{aligned}$$

since $\frac{4-2}{4}\cdot \frac{3}{4}-\frac{1}{2}\ge -\frac{1}{8}$ and $(4-2)(1-\frac{3}{4}) - \frac{1}{2}\ge -\frac{1}{4}$. Similarly for the third derivative with $\theta =\frac{1}{2}$ we get

C.9 $$\begin{aligned}&\partial _x^3 :{\mathbb {X}}_T \rightarrow L^{\frac{8}{3}}(0,T;L^4), \nonumber \\&\text {with the estimate } \Vert \partial _x^3 f \Vert _{L^{\frac{8}{3}}(0,T;L^4)}\le {C} \Vert f \Vert _{{\mathbb {X}}_T} \quad \text { for all }f\in {}_{0}{{\mathbb {X}}_{T}}. \end{aligned}$$

Thus, condition (ii) a) in Lemma C.5 is satisfied for the fist *m* factors in (C.6) by (C.7), whereas for the remaining factors condition (ii) b) holds. More precisely, for $j=m+1$ choosing $k=m+2$ works and conversely $j=m+2,k=m+1$, using Remark C.6.

The case $(a,b)=(3,0)$ can be treated similarly, using Hölder to obtain

$$\begin{aligned}&\Vert \varphi (\partial _x f_1, \ldots , \partial _x f_m, \partial _x^2 g_1, \partial _x^2 g_2, \partial _x^2 g_3) \Vert _{L^2(0,T;L^2)} \\ {}&\quad \le C\prod _{j=1}^m\Vert \partial _x f_j \Vert _{\infty } \prod _{j=1}^{3}\Vert \partial _x^2g_j \Vert _{L^{6}(0,T;L^{6})}, \end{aligned}$$

and then Proposition B.3 (i) with $k=2$, $\theta =\frac{2}{3}$ to get

C.10 $$\begin{aligned}&\partial _x^2 :{\mathbb {X}}_T \rightarrow L^6(0,T;L^6), \nonumber \\&\text {with the estimate } \Vert \partial _x^2 f \Vert _{L^6(0,T;L^6)}\le {C} \Vert f \Vert _{{\mathbb {X}}_T}\quad \text { for all }f\in {}_{0}{{\mathbb {X}}_{T}}. \end{aligned}$$

Case (ii): First, we have the following basic estimate

$$\begin{aligned} \left\| \int \nolimits _I \Phi (f)\,\!\textrm{d}x-\int \nolimits _{I}\Phi ({\tilde{f}})\,\!\textrm{d}x \right\| _{L^2(0,T)} \le \Vert \Phi (f)-\Phi ({\tilde{f}}) \Vert _{L^2(0,T;L^1)}. \end{aligned}$$

It hence suffices to show that ${\mathbb {X}}_T\rightarrow L^2(0,T;L^1), f\mapsto \Phi (f)$ is a *q*-contraction. To that end, we use Lemma C.5 with $Z=L^1, S={{\,\textrm{Id}\,}}$.

If $(a,b)=(0,2)$, we have by Hölder’s inequality

C.11 $$\begin{aligned}&\Vert \varphi (\partial _xf_1, \dots \partial _x f_m, \partial _x^3h_1, \partial _x^3h_2) \Vert _{L^2(0,T;L^1)} \le C \prod _{j=1}^m\Vert \partial _x f_j \Vert _{\infty } \prod _{j=1}^{2}\Vert \partial _x^{3}h_j \Vert _{L^{4}(0,T;L^2)}. \end{aligned}$$

Now, using Proposition B.3 (i) with $k=3$ and $\theta =1$, we have

C.12 $$\begin{aligned}&\partial _x^3 :{\mathbb {X}}_T \rightarrow L^{4}(0,T;L^2(I;{\mathbb {R}}^d))\nonumber \\&\text {with the estimate } \Vert \partial _x^3 f \Vert _{L^4(0,T;L^2)}\le {C} \Vert f \Vert _{{\mathbb {X}}_T} \quad \text { for all }f\in {}_{0}{{\mathbb {X}}_{T}}. \end{aligned}$$

Consequently, the last two factors in (C.11) satisfy condition (i) and (ii) b) in Lemma C.5, cf. Remark C.6. For the first *m* factors, we may once again use (C.7) to deduce that conditions (i) and (ii) a) in Lemma C.5 are satisfied.

If $(a,b)=(2,1)$, we proceed similarly, first using Hölder to get

$$\begin{aligned}&\Vert \varphi (\partial _x f_1, \dots \partial _xf_m,\partial _x^2g_1, \partial _x^2g_2, \partial _x^3h) \Vert _{L^2(0,T;L^1)} \\&\quad \le C \prod _{j=1}^m\Vert \partial _x f_j \Vert _{\infty } \prod _{j=1}^{2}\Vert \partial _x^2g_j \Vert _{L^{8}(0,T;L^4)}\Vert \partial _x^3 h \Vert _{L^4(0,T;L^2)}, \end{aligned}$$

and then applying (C.7), (C.8) and (C.12). For $(a,b)=(4,0)$, we may apply Hölder’s inequality to obtain

$$\begin{aligned}&\Vert \varphi (\partial _xf_1, \dots \partial _xf_m,\partial _x^2g_1, \partial _x^2g_2, \partial _x^2g_3,\partial _x^2 g_4) \Vert _{L^2(0,T;L^1)} \\&\quad \le C\prod _{j=1}^m\Vert \partial _x f_j \Vert _{\infty } \prod _{j=1}^{4}\Vert \partial _x^2g_j \Vert _{L^{8}(0,T;L^4)}, \end{aligned}$$

and then use (C.7) and (C.8).

Case (iii): Again, we use Lemma C.5, now with $Z=({\mathbb {R}}^d)^2$ and $S={{\,\textrm{tr}\,}}_{\partial I}$. If $(a,b)=(1,1)$ we obtain by Hölder’s inequality

C.13 $$\begin{aligned}&\Vert {{\,\textrm{tr}\,}}_{\partial I} \varphi (\partial _xf_1, \dots , \partial _xf_m, \partial _x^2g,\partial _x^3h) \Vert _{L^2(0,T;({\mathbb {R}}^d)^{2})} \nonumber \\&\quad \le C\prod _{j=1}^m\Vert {{\,\textrm{tr}\,}}_{\partial I}\partial _x f_j \Vert _{\infty } \Vert {{\,\textrm{tr}\,}}_{\partial I}\partial _x^2g \Vert _{L^8(0,T;({\mathbb {R}}^d)^2)}\Vert {{\,\textrm{tr}\,}}_{\partial I}\partial _x^3 h \Vert _{L^{\frac{8}{3}}(0,T;({\mathbb {R}}^d)^2)}. \end{aligned}$$

Note that by Proposition B.3 (ii), we have

C.14 $$\begin{aligned}&{{\,\textrm{tr}\,}}_{\partial I}\partial _x^2 :{\mathbb {X}}_T \rightarrow L^{8}(0,T;({\mathbb {R}}^d)^2), \nonumber \\&\text {with the estimate } \Vert {{\,\textrm{tr}\,}}_{\partial I}\partial _x^2 f \Vert _{L^{8}(0,T;({\mathbb {R}}^d)^2)}\le {C} \Vert f \Vert _{{\mathbb {X}}_T}\quad \text { for all }f\in {}_{0}{{\mathbb {X}}_{T}}, \end{aligned}$$

whereas for the third derivative, we obtain

C.15 $$\begin{aligned}&{{\,\textrm{tr}\,}}_{\partial I}\partial _x^3 :{\mathbb {X}}_T \rightarrow L^{\frac{8}{3}}(0,T;({\mathbb {R}}^d)^2),\nonumber \\&\text {with the estimate } \Vert {{\,\textrm{tr}\,}}_{\partial I}\partial _x^3 f \Vert _{L^{\frac{8}{3}}(0,T;({\mathbb {R}}^d)^2)}\le {C} \Vert f \Vert _{{\mathbb {X}}_T}\quad \text { for all }f\in {}_{0}{{\mathbb {X}}_{T}}. \end{aligned}$$

As in cases (i) and (ii), we then use the mapping properties and the estimates in (C.7), (C.14) and (C.15) together with Remark C.6 to verify that the assumptions of Lemma C.5 are satisfied.

If $(a,b)=(3,0)$, we proceed similarly, first using Hölder to obtain

$$\begin{aligned}&\Vert {{\,\textrm{tr}\,}}_{\partial I} \varphi (\partial _xf_1, \dots , \partial _xf_m, \partial _x^2g_1, \partial _x^2g_2, \partial _x^2g_3) \Vert _{L^2(0,T;({\mathbb {R}}^d)^{2})} \\&\quad \le C\prod _{j=1}^m\Vert \partial _x f_j \Vert _{\infty } \prod _{j=1}^{3}\Vert \partial _x^2g_j \Vert _{L^6(0,T;({\mathbb {R}}^d)^2)}, \end{aligned}$$

and then (C.7) for the first-order terms and Proposition B.3 (ii) with $k=2$, yielding

$$\begin{aligned}&{{\,\textrm{tr}\,}}_{\partial I}\partial _x^2 :{\mathbb {X}}_T\rightarrow L^{6}(0,T;({\mathbb {R}}^d)^2), \\&\text {with the estimate } \Vert {{\,\textrm{tr}\,}}_{\partial I}\partial _x^2 f \Vert _{L^{6}(0,T;({\mathbb {R}}^d)^2)}\le {C} \Vert f \Vert _{{\mathbb {X}}_T}\quad \text { for all }f\in {}_{0}{{\mathbb {X}}_{T}}. \end{aligned}$$

Case (iv): Let $q\in (0,1)$. For $f, {\tilde{f}}\in {{\bar{B}}_{T,M}}$, we have

$$\begin{aligned}&|(\gamma _0^{-4}-\gamma ^{-4})\partial _x^4 f - (\gamma _0^{-4}-{\tilde{\gamma }}^{-4})\partial _x^4 {\tilde{f}}| \le |\gamma _0^{-4}-\gamma ^{-4}| |\partial _x^4 f-\partial _x^4 {\tilde{f}}| \\&\quad + |{\tilde{\gamma }}^{-4}-\gamma ^{-4}||\partial _x^4 {\tilde{f}}|. \end{aligned}$$

Thus, we may estimate

C.16 $$\begin{aligned}&\Vert (\gamma _0^{-4}-\gamma ^{-4})\partial _x^4 f - (\gamma _0^{-4}-{\tilde{\gamma }}^{-4})\partial _x^4 {\tilde{f}} \Vert _{L^2(0,T;L^2)}\nonumber \\&\quad \le \Vert \gamma _0^{-4}-\gamma ^{-4} \Vert _{\infty }\Vert \partial _x^4 f-\partial _x^4 {\tilde{f}} \Vert _{L^2(0,T;L^2)} + \Vert {\tilde{\gamma }}^{-4}-\gamma ^{-4} \Vert _{\infty } \Vert \partial _x^4 {\tilde{f}} \Vert _{L^2(0,T;L^2)}. \end{aligned}$$

For the first term, we use the mean value theorem and Lemma 2.2 to conclude

C.17 $$\begin{aligned} |\gamma _0(x)^{-4}-\gamma (t,x)^{-4}| \le 4 \left( \frac{\inf _{I}\gamma _0}{2} \right) ^{-5}|\partial _x f_0(x) -\partial _x f(t,x)|. \end{aligned}$$

Consequently, using Proposition B.1 (ii) we may estimate

$$\begin{aligned} \Vert \gamma _0^{-4}-\gamma ^{-4} \Vert _{\infty }&\le C(\gamma _0) \sup _{(t,x)\in [0,T]\times I} |\partial _x f(0,x)-\partial _xf(t,x)| \\&\le C(\gamma _0) \sup _{(t,x)\in [0,T]\times I} \Vert f(0)-f(t) \Vert _{{\mathcal {C}}^{1+\alpha }(I;{\mathbb {R}}^d)} \\&\le C(\gamma _0) \sup _{(t,x)\in [0,T]\times I} t^{\alpha } \Vert f \Vert _{{\mathcal {C}}^{\alpha }([0,T]; {\mathcal {C}}^{1+\alpha }(I;{\mathbb {R}}^d))} \\&\le C(\gamma _0) T^{\alpha }\left( \Vert f-{\bar{f}} \Vert _{{\mathcal {C}}^{\alpha }([0,T]; {\mathcal {C}}^{1+\alpha }(I;{\mathbb {R}}^d))} +\Vert {\bar{f}} \Vert _{{\mathcal {C}}^{\alpha }([0,T]; {\mathcal {C}}^{1+\alpha }(I;{\mathbb {R}}^d))}\right) \\&\le {C(\gamma _0)} T^{\alpha } \left( \Vert f-{\bar{f}} \Vert _{{\mathbb {X}}_T}+\Vert {\bar{f}} \Vert _{{\mathcal {C}}^{\alpha }([0,T]; {\mathcal {C}}^{1+\alpha }(I;{\mathbb {R}}^d))}\right) \\&\le {C({\bar{f}})} T^{\alpha }. \end{aligned}$$

Combined with the simple estimate $\Vert \partial _x^4 f -\partial _x^{4}{\tilde{f}} \Vert _{L^2(0,T;L^2)}\le \Vert f-{\tilde{f}} \Vert _{{\mathbb {X}}_T}$ this yields a $\frac{q}{2}$-contraction estimate for the first part of (C.16), taking ${T=T(q, {\bar{f}})\in (0,1]}$ small enough. For the remaining part, we use (C.17) with $\gamma _0$ replaced by ${\tilde{\gamma }}$ to conclude

$$\begin{aligned} \Vert {\tilde{\gamma }}^{-4}-\gamma ^{-4} \Vert _{\infty }\le 4 \left( \inf _{I}\frac{\gamma _0}{2}\right) ^{-5} \Vert \partial _x f-\partial _x {\tilde{f}} \Vert _{\infty } \le {C({\bar{f}})} T^{\alpha }\Vert f-{\tilde{f}} \Vert _{{\mathbb {X}}_T}, \end{aligned}$$

and $\Vert \partial _x^4 f \Vert _{L^2(0,T;L^2)} \le \Vert f-{\bar{f}} \Vert _{{\mathbb {X}}_T} + \Vert \partial _x^4 {\bar{f}} \Vert _{L^2(0,T;L^2)} \le {C({\bar{f}})}$. Consequently, if ${T=T(q, {\bar{f}})\in (0,1]}$ is small enough, the second part of (C.16) is a $\frac{q}{2}$-contraction. $\square$

It is not difficult to see that the statement of Proposition C.4 remains true if one allows multiplication by powers of the arc-length element.

### Corollary C.7

Let $q\in (0,1),\ell \in {\mathbb {N}}$, $\Phi \in {\mathcal {A}}^{(a,b)}$. For ${T=T(q,\ell ) \in (0,1]}$, ${M=}$ ${M(q, \ell )\in (0,1]}$ small enough, each of the following maps is a well-defined *q*-contraction.

(i) $${{\bar{B}}_{T,M}}\rightarrow L^2(0,T;L^2), f\mapsto \gamma ^{-\ell }\Phi (f)$$, if $$(a,b)=(1,1)$$ or $$(a,b)=(3,0)$$.
(ii) ${{\bar{B}}_{T,M}} \rightarrow L^2(0,T), f\mapsto \int \nolimits _{I} \gamma ^{-\ell }\Phi (f)\!\textrm{d}x$, if $(a,b)=(0,2), (a,b)=(2,1)$ or $(a,b)=(4,0)$.
(iii) ${{\bar{B}}_{T,M}} \rightarrow L^2(0,T({\mathbb {R}}^{d})^2), f\mapsto {{\,\textrm{tr}\,}}_{\partial I}\gamma ^{-\ell } \Phi (f)$, if $(a,b)=(1,1)$ or $(a,b)=(3,0)$.

### Proof

Well-definedness: By Lemma 2.2 we can estimate $|\gamma ^{-\ell } \Phi (f)|\le \frac{\inf \gamma _0}{2} |\Phi (f)|$ for all $T,M>0$ small enough. Thus $f\mapsto \gamma ^{-\ell }\Phi (f)$ maps into the correct space by Lemma C.5.

Contraction: Let $q\in (0,1)$ and let $f, {\tilde{f}}\in {{\bar{B}}_{T,M}}$. For the first case, taking $T,M>0$ small enough, we have

$$\begin{aligned}&\Vert \gamma ^{-\ell }\Phi (f)-{\tilde{\gamma }}^{-\ell }\Phi ({\tilde{f}}) \Vert _{L^2(0,T;L^2)} \\&\quad \le \Vert \gamma ^{-\ell }-{\tilde{\gamma }}^{-\ell } \Vert _{\infty } \Vert \Phi (f) \Vert _{L^2(0,T;L^2)} + \Vert {\tilde{\gamma }}^{-\ell } \Vert _{\infty } \Vert \Phi (f)-\Phi ({\tilde{f}}) \Vert _{L^2(0,T;L^2)} \\&\quad \le \Vert \gamma ^{-\ell }-{\tilde{\gamma }}^{-\ell } \Vert _{\infty } \left( \Vert \Phi (f)- \Phi ({\bar{f}}) \Vert _{L^2(0,T;L^2)}+\Vert \Phi ({\bar{f}}) \Vert _{L^2(0,T;L^2)}\right) \\&\quad \quad + \left( \Vert {\tilde{\gamma }}^{-\ell }-{\bar{\gamma }}^{-\ell } \Vert _{\infty }+\Vert {\bar{\gamma }}^{-\ell } \Vert _{\infty }\right) \Vert \Phi (f)-\Phi ({\tilde{f}}) \Vert _{L^2(0,T;L^2)} \\&\quad \le {C({\bar{f}})}\Vert \gamma ^{-\ell }-{\tilde{\gamma }}^{-\ell } \Vert _{\infty }+ \left( \Vert {\tilde{\gamma }}^{-\ell }-{\bar{\gamma }}^{-\ell } \Vert _{\infty }+\Vert {\bar{\gamma }}^{-\ell } \Vert _{\infty }\right) q_2\Vert f-{\tilde{f}} \Vert _{{\mathbb {X}}_T} \end{aligned}$$

using Proposition C.4 for $q_2\in (0,1)$ to be chosen. With similar estimates one finds

$$\begin{aligned}&\left\| \int \nolimits _I \gamma ^{-\ell }\Phi (f)\!\textrm{d}x - \int \nolimits _I{\tilde{\gamma }}^{-\ell }\Phi ({\tilde{f}})\!\textrm{d}x \right\| _{L^2(0,T)} \\&\quad \le {C({\bar{f}})}\Vert \gamma ^{-\ell }-{\tilde{\gamma }}^{-\ell } \Vert _{\infty }+ \left( \Vert {\tilde{\gamma }}^{-\ell }-{\bar{\gamma }}^{-\ell } \Vert _{\infty }+\Vert {\bar{\gamma }}^{-\ell } \Vert _{\infty }\right) q_2\Vert f-{\tilde{f}} \Vert _{{\mathbb {X}}_T} \end{aligned}$$

and

$$\begin{aligned}&\Vert {{\,\textrm{tr}\,}}_{\partial I}\gamma ^{-\ell }\Phi (f) -{{\,\textrm{tr}\,}}_{\partial I}{\tilde{\gamma }}^{-\ell }\Phi ({\tilde{f}}) \Vert _{L^2(0,T;({\mathbb {R}}^d)^2)} \\&\quad \le {C({\bar{f}})}\Vert \gamma ^{-\ell }-{\tilde{\gamma }}^{-\ell } \Vert _{\infty }+ \left( \Vert {\tilde{\gamma }}^{-\ell }-{\bar{\gamma }}^{-\ell } \Vert _{\infty }+\Vert {\bar{\gamma }}^{-\ell } \Vert _{\infty }\right) q_2\Vert f-{\tilde{f}} \Vert _{{\mathbb {X}}_T}. \end{aligned}$$

We now prove that for any $q\in (0,1)$ the map ${{\bar{B}}_{T,M}}\rightarrow L^{\infty }((0,T)\times I)$, $f\mapsto \gamma ^{-\ell }$ is a *q*-contraction for $T,M>0$ small enough. We find as in (C.17)

$$\begin{aligned} \Vert \gamma ^{-\ell }-{\tilde{\gamma }}^{-\ell } \Vert _{\infty }&\le {C(\ell , {\bar{f}})} \Vert f-{\tilde{f}} \Vert _{{\mathcal {C}}^0([0,T];{\mathcal {C}}^{1}(I;{\mathbb {R}}^d))}\\&\le {C(\ell , {\bar{f}})} T^{\alpha }\Vert f-{\tilde{f}} \Vert _{{\mathbb {X}}_T}\le \frac{q}{2} \Vert f-{\tilde{f}} \Vert _{{\mathbb {X}}_T}, \end{aligned}$$

for ${T=T(q, \ell , {\bar{f}})\in (0,1]}$ small enough using Proposition B.1 (ii) and the fact that $f(0)={\tilde{f}}(0)=f_0$. Thus, we find

$$\begin{aligned}&{C({\bar{f}})}\Vert \gamma ^{-\ell }-{\tilde{\gamma }}^{-\ell } \Vert _{\infty }+ \left( \Vert {\tilde{\gamma }}^{-\ell }-{\bar{\gamma }}^{-\ell } \Vert _{\infty }+\Vert {\bar{\gamma }}^{-\ell } \Vert _{\infty }\right) q_2\Vert f-{\tilde{f}} \Vert _{{\mathbb {X}}_T} \\&\quad \le \frac{q}{2}\Vert f-{\tilde{f}} \Vert _{{\mathbb {X}}_T} + \left( M + \Vert {\bar{\gamma }}^{-\ell } \Vert _{\infty }\right) q_2 \Vert f-{\tilde{f}} \Vert _{{\mathbb {X}}_T} \le q\Vert f-{\tilde{f}} \Vert _{{\mathbb {X}}_T}, \end{aligned}$$

choosing first ${q_2 = q_2 (q, \ell , {\bar{f}}) \in (0,1)}$ sufficiently small and passing to a smaller ${T = T(q, \ell , {\bar{f}})}$ and ${M= M(q, \ell , {\bar{f}})\in (0,1]}$ if necessary. $\square$

### Proof of Lemma 2.4

First, taking ${T=T({\bar{f}}),M\in (0,1]}$ small enough such that Lemmas 2.2 and 2.3 hold, all terms are defined almost everywhere. We observe that ${\mathcal {F}}(f)$ is a sum of terms as in Corollary C.7 (i) and Proposition C.4 (iv) by (2.2), hence well-defined and a *q*-contraction for all $q\in (0,1)$, if ${T=T(q, {\bar{f}})}$, ${M=M(q, {\bar{f}})\in (0,1]}$ are small enough.

For $\Lambda$ we need to do one additional estimate. For $f\in {{\bar{B}}_{T,M}}$ and $T,M>0$ the scalar-valued function $\lambda$ is in $L^{2}(0,T)$, since by Lemma 2.3 the energy ${\mathcal {E}}(f)$ in the denominator of $\lambda$ (cf. Sect. 2.1) is bounded from below uniformly in *t*, whereas the nominator *N*(*f*) is in $L^2(0,T)$ by Corollary C.7 (ii) and (iii) and by the explicit formulas in Lemma A.2 and (2.1). The term $\vec {\kappa }_{{f}}$ is in $L^{\infty }(0,T)$ by the embedding ${\mathbb {X}}_T\hookrightarrow BUC([0,T]; W^{2,2}(I;{\mathbb {R}}^d))$, cf. Proposition B.1 (i),(B.1) and Proposition A.1.

Now, the crucial step is the proof of the contraction estimate for $\Lambda$. To that end, let $f, {\tilde{f}}\in {{\bar{B}}_{T,M}}$. Then, writing $\lambda (f)=\frac{N(f)}{2{\mathcal {E}}(f)}$ as in Sect. 2.1, we find for almost every (*t*, *x*)

C.18 $$\begin{aligned}&|\lambda (f)(t)\vec {\kappa }_f(t,x) - \lambda ({\tilde{f}})(t)\vec {\kappa }_{{{\tilde{f}}}}(t,x)|\nonumber \\&\quad \le |\lambda (f)(t)-\lambda ({\tilde{f}})(t)| |\vec {\kappa }_{{f}}(t,x)| + |\lambda ({\tilde{f}})(t)| |\vec {\kappa }_f(t,x)-\vec {\kappa }_{{\tilde{f}}}(t,x)| \nonumber \\&\quad \le \frac{1}{2{\mathcal {E}}(f(t)){\mathcal {E}}({\tilde{f}}(t))} |N(f)(t)| |{\mathcal {E}}(f(t))-{\mathcal {E}}({\tilde{f}}(t))||\vec {\kappa }_{{f}}(t,x)|\nonumber \\&\quad \quad + \frac{1}{2{\mathcal {E}}(f(t))}|N(f)(t)-N({\tilde{f}})(t)||\vec {\kappa }_f(t,x)| + |\lambda (f)(t)| |\vec {\kappa }_{{f}}(t,x)-\vec {\kappa }_{{{\tilde{f}}}}(t,x)| \nonumber \\&\quad \le C(f_0) |N(f)(t)| |{\mathcal {E}}(f(t))-{\mathcal {E}}({\tilde{f}}(t))||\vec {\kappa }_{{f}}(t,x)| \nonumber \\&\quad \quad + C(f_0)|N(f)(t)-N({\tilde{f}})(t)||\vec {\kappa }_f(t,x)| + |\lambda (f)(t)| |\vec {\kappa }_{{f}}(t,x)-\vec {\kappa }_{{{\tilde{f}}}}(t,x)|, \end{aligned}$$

using that by Lemma 2.3 the elastic energy is bounded from below. Taking the $L^2L^2$-norm in (C.18), we are left with three terms. The first one is

C.19 $$\begin{aligned}&\Vert |N(f)| |{\mathcal {E}}(f)-{\mathcal {E}}({\tilde{f}})||\vec {\kappa }_{{f}}| \Vert _{L^2(0,T;L^2)}\nonumber \\&\quad \le \Vert N(f) \Vert _{L^2(0,T)} \Vert {\mathcal {E}}(f)-{\mathcal {E}}({\tilde{f}}) \Vert _{L^{\infty }(0,T)} \Vert \vec {\kappa }_f \Vert _{L^{\infty }(0,T;L^2)}. \end{aligned}$$

Now, note that *N*(*f*) is a sum of terms as in Corollary C.7 (ii) and (iii) by (2.1) and the explicit formulas in Lemma A.2. Therefore, for any $q\in (0,1)$, we have

C.20 $$\begin{aligned} \Vert N(f) - N({\tilde{f}}) \Vert _{L^2(0,T)} \le q \Vert f-{\tilde{f}} \Vert _{{\mathbb {X}}_{T}}, \end{aligned}$$

if we take ${T=T(q, {\bar{f}}),M=M(q, {\bar{f}})\in (0,1]}$ small enough. In particular, we can assume that $f\mapsto N(f)$ is 1-Lipschitz.

For the elastic energy term, note that ${\mathcal {E}}$ is analytic, hence ${\mathcal {C}}^{1}$ on the space of $W^{2,2}$-immersions, cf. Proposition 4.4, in particular it is locally Lipschitz continuous in a neighbourhood of $f_0\in W^{2,2}_{Imm}(I;{\mathbb {R}}^d)$. Hence, there exists $C(f_0)>0$ such that $|{\mathcal {E}}(h)-{\mathcal {E}}({\tilde{h}})|\le C(f_0)\Vert h-{\tilde{h}} \Vert _{W^{2,2}(I;{\mathbb {R}}^d)}$ for all *h* and ${\tilde{h}}$ satisfying $\Vert h-f_0 \Vert _{W^{2,2}}<\delta$ and $\Vert {\tilde{h}}-f_0 \Vert _{W^{2,2}}\le \delta$.

By Proposition B.1(i), we have ${}_{0}{{\mathbb {X}}}_T \hookrightarrow BUC(0,T;W^{2,2})$ with operator norm independent of $T\in (0,1]$. Consequently, we have

$$\begin{aligned} \Vert f(t)-f_0 \Vert _{W^{2,2}}&\le \Vert f(t)-{\bar{f}}(t) \Vert _{W^{2,2}}+ \Vert {\bar{f}}(t)-f_0 \Vert _{W^{2,2}} \\&\le {C}M +\Vert {\bar{f}}(t)-{\bar{f}}(0) \Vert _{W^{2,2}} \le \delta \end{aligned}$$

for ${ T=T({\bar{f}}), M\in (0,1]}$ small enough, and similarly $\Vert {\tilde{f}}(t)-f_0 \Vert _{W^{2,2}}\le \delta$. But then, using Proposition B.1(i), we have the estimate

C.21 $$\begin{aligned} \Vert {\mathcal {E}}(f)-{\mathcal {E}}({\tilde{f}}) \Vert _{L^{\infty }(0,T)}\le C(f_0) \Vert f-{\tilde{f}} \Vert _{L^{\infty }(0,T;W^{2,2})} \le {C({\bar{f}})} \Vert f-{\tilde{f}} \Vert _{{\mathbb {X}}_T}. \end{aligned}$$

For the curvature term $\vec {\kappa }_f$, note that $W^{2,2}_{Imm}(I;{\mathbb {R}}^d)\rightarrow L^2(I;{\mathbb {R}}^d), f\mapsto \vec {\kappa }_{f} = \partial _{s_f}^2 f$ is analytic (cf. Proposition 4.4), in particular Lipschitz continuous near $f_0$. The same argument as above yields

C.22 $$\begin{aligned} \Vert \vec {\kappa }_{f}-\vec {\kappa }_{{\tilde{f}}} \Vert _{L^{\infty }(0,T;L^2)}\le C(f_0) \Vert f-{\tilde{f}} \Vert _{L^{\infty }(0,T;W^{2,2})} \le {C({\bar{f}})}\Vert f-{\tilde{f}} \Vert _{{\mathbb {X}}_T}. \end{aligned}$$

Now, we estimate

C.23 $$\begin{aligned} \Vert \vec {\kappa }_{f} \Vert _{L^{\infty }(0,T;L^{2})}&\le \Vert \vec {\kappa }_{f}-\vec {\kappa }_{{\bar{f}}} \Vert _{L^{\infty }(0,T;L^2)} + \Vert \vec {\kappa }_{{\bar{f}}} \Vert _{L^{\infty }(0;T;L^2)}\nonumber \\&\le {C({\bar{f}})} \Vert f-{\bar{f}} \Vert _{{\mathbb {X}}_T}+\Vert \vec {\kappa }_{{\bar{f}}} \Vert _{L^{\infty }(0;T;L^2)}\le {C({\bar{f}})} \end{aligned}$$

and using (C.20), we obtain the bound

C.24 $$\begin{aligned} \Vert N(f) \Vert _{L^2(0,T)}&\le \Vert N(f)-N({\bar{f}}) \Vert _{L^2(0,T)}+\Vert N({\bar{f}}) \Vert _{L^2(0,T)}\nonumber \\&\le \Vert f-{\bar{f}} \Vert _{{\mathbb {X}}_T}+\Vert N({\bar{f}}) \Vert _{L^2(0,T)} \le M+\Vert N({\bar{f}}) \Vert _{L^2(0,T)}. \end{aligned}$$

If we now combine (C.21), (C.23) and (C.24), we obtain from (C.19)

$$\begin{aligned}&C(f_0)\Vert |N(f)| |{\mathcal {E}}(f)-{\mathcal {E}}({\tilde{f}})||\vec {\kappa }_{{f}}| \Vert _{L^2(0,T;L^2)} \\&\quad \le \left( M+\Vert N({\bar{f}}) \Vert _{L^2(0,T)}\right) {C({\bar{f}})}\Vert f-{\tilde{f}} \Vert _{{\mathbb {X}}_T}\le \frac{q}{4}\Vert f-{\tilde{f}} \Vert _{{\mathbb {X}}_T} \end{aligned}$$

if we take ${T=T(q,{\bar{f}}), M=M(q,{\bar{f}})\in (0,1]}$ small enough.

For the second term in (C.18), using (C.20) and (C.23) we have

$$\begin{aligned} \begin{aligned} C(f_0)\Vert N(f)(t)-N({\tilde{f}})(t) \Vert _{L^2(0,T)}\Vert \vec {\kappa }_f(t,x) \Vert _{L^{\infty }(0,T;L^2)}&\le {C({\bar{f}})}q_2\Vert f-{\tilde{f}} \Vert _{{\mathbb {X}}_T} \\&\le \frac{q}{4}\Vert f-{\tilde{f}} \Vert _{{\mathbb {X}}_T}, \end{aligned} \end{aligned}$$

taking ${q_2=q_2(q, {\bar{f}})\in (0,1)}$ small enough and possibly reducing ${T=T(q,{\bar{f}})}$, ${M=}$ ${M(q, {\bar{f}})\in (0,1]}$ if necessary.

For the last term in (C.18), using Lemma 2.3, (C.24) and (C.22), we have

$$\begin{aligned}&\left\| \frac{N(f)}{{\mathcal {E}}(f)} \right\| _{L^{2}(0,T)}\Vert \vec {\kappa }_f-\vec {\kappa }_{{\tilde{f}}} \Vert _{L^{\infty }(0,T;L^2)} \\&\quad \le \frac{3}{{\mathcal {E}}(f_0)} \left( M+\Vert N({\bar{f}}) \Vert _{L^2(0,T)}\right) {C({\bar{f}})}\Vert f-{\tilde{f}} \Vert _{{\mathbb {X}}_T} \le \frac{q}{4}\Vert f-{\tilde{f}} \Vert _{{\mathbb {X}}_T}. \end{aligned}$$

taking ${M=M(q,{\bar{f}}),T=T(q,{\bar{f}})\in (0,1]}$ small enough. All in all, we have now shown that taking ${T=T(q,{\bar{f}}), M=M(q,{\bar{f}})\in (0,1]}$ small enough, we have

$$\begin{aligned} \Vert \Lambda (f)-\Lambda ({\tilde{f}}) \Vert _{L^{2}(0,T;L^2)}=\Vert \lambda (f)\vec {\kappa }_f-\lambda ({\tilde{f}})\vec {\kappa }_{{\tilde{f}}} \Vert _{L^2(0,T;L^2)}\le \frac{3q}{4}\Vert f-{\tilde{f}} \Vert _{{\mathbb {X}}_T}, \end{aligned}$$

which proves that $$\Lambda :{{\bar{B}}_{T,M}}\rightarrow L^2(0,T;L^2)$$ is a $$\frac{3q}{4}$$-contraction. Reducing $${T=} $$ $$ {T(q,{\bar{f}})}\in (0,1]$$, $${M=M(q,{\bar{f}}) \in (0,1]}$$ if necessary, we may assume that $${\mathcal {F}}$$ is a $$\frac{q}{4}$$-contraction; hence, $${\mathcal {N}}:{{\bar{B}}_{T,M}}\rightarrow {\mathbb {Y}}_T^{1}$$ is a *q*-contraction for $$T=T(q, {\bar{f}}),M=M(q,{\bar{f}})\in (0,1]$$ small enough. $$\square $$

## Appendix D: A gluing lemma for reparametrizations

In Theorem 4.1, we used the fact that two smooth reparametrizations can be interpolated by another smooth reparametrization. We state this gluing result here in a slightly more general form for possible future reference.

### Lemma D.1

Let $0<t_1<t_2<T$ and $\Phi _1:[0,t_2]\times I\rightarrow I$, $\Phi _2:[t_1, T]\times I\rightarrow I$ be smooth families of reparametrizations, such that $\Phi _i(t, \cdot )$ is strictly increasing for all suitable *t* and $i=1,2$. Then, there exists a smooth family of strictly increasing reparametrizations $\Psi :[0,T]\times I\rightarrow I$ satisfying

$$\begin{aligned} \Psi (t,x)&= \Phi _1(t,x), \quad \text { for all }0\le t\le t_1, x\in I\\ \Psi (t,x)&= \Phi _2(t,x), \quad \text { for all }t_2\le t\le T, x\in I. \end{aligned}$$

### Proof

Let $\delta >0$ be sufficiently small and $\eta :[0,T]\rightarrow {\mathbb {R}}, 0\le \eta \le 1$ be a smooth cut-off function, satisfying

$$\begin{aligned} \eta (t) = \left\{ \begin{array}{ll} 1, &{}\text { for } 0\le t\le t_1+\delta \\ 0, &{}\text { for } t\ge t_2-\delta . \end{array}\right. \end{aligned}$$

Then it is not difficult to check that the function $\Psi :[0,T]\times I\rightarrow {\mathbb {R}}$ given by

$$\begin{aligned} \Psi (t,x) :=\left\{ \begin{array}{ll} \Phi _1(t,x)&{} \text { for }0\le t\le t_1, x\in I\\ \Phi _1(t,x) \eta (t) + \Phi _2(t,x)(1-\eta (t)), &{}\text { for } t\in [t_1, t_2], x\in I\\ \Phi _2(t,x) &{} \text { for }t_2\le t\le T, x\in I \end{array}\right. \end{aligned}$$

is smooth and satisfies all the desired properties. $\square$
